# Use of safety-engineered devices by healthcare workers for intravenous and/or phlebotomy procedures in healthcare settings: a systematic review and meta-analysis

**DOI:** 10.1186/s12913-016-1705-y

**Published:** 2016-09-01

**Authors:** Rami A. Ballout, Batoul Diab, Alain C. Harb, Rami Tarabay, Selma Khamassi, Elie A. Akl

**Affiliations:** 1 Faculty of Medicine, American University of Beirut, Beirut, Lebanon; 2 Lebanese University, Beirut, Lebanon; 3 World Health Organization, Geneva, Switzerland; 4 Department of Internal Medicine, American University of Beirut Medical Center, Riad-El-Solh, P.O. Box: 11-0236, Beirut, 1107 2020 Lebanon

**Keywords:** Systematic review, Healthcare workers, Healthcare setting, Needle-stick injuries, Safety-engineered devices, Intravenous, Phlebotomy, Meta-analysis, Blood-borne pathogens

## Abstract

**Background:**

The acquisition of needle-stick injuries (NSI) in a healthcare setting poses an occupational hazard of transmitting blood-borne pathogens from patients to healthcare workers (HCWs). The objective of this study was to systematically review the evidence about the efficacy and safety of using safety-engineered intravenous devices and safety-engineered phlebotomy devices by HCWs.

**Methods:**

We included randomized and non-randomized studies comparing safety-engineered devices to conventional/standard devices that lack safety features for delivering intravenous injections and/or for blood-withdrawal procedures (phlebotomy). The outcomes of interest included NSI rates, and blood-borne infections rates among HCWs and patients. We conducted an extensive literature search strategy using the OVID interface in October 2013. We followed the standard methods for study selection and data abstraction. When possible, we conducted meta-analyses using a random-effects model. We used the GRADE methodology to assess the quality of evidence by outcome.

**Results:**

We identified twenty-two eligible studies: Twelve assessed safety-engineered devices for intravenous procedures, five for phlebotomy procedures, and five for both. Twenty-one of those studies were observational while one was a randomized trial. All studies assessed the reduction in NSIs among HCWs. For safety-engineered intravenous devices, the pooled relative risk for NSI per HCW was 0.28 [0.13, 0.59] (moderate quality evidence). The pooled relative risk for NSI per device used or procedure performed was 0.34 [0.08,1.49] (low quality evidence). For safety-engineered phlebotomy devices, the pooled relative risk for NSI per HCW was 0.57 [0.38, 0.84] (moderate quality evidence). The pooled relative risk for NSI per device used or procedure performed was 0.53 [0.43,0.65] (moderate quality evidence). We identified no studies assessing the outcome of blood-borne infections among healthcare workers or patients.

**Conclusion:**

There is moderate-quality evidence that the use of safety-engineered devices in intravenous injections and infusions, and phlebotomy (blood-drawing) procedures reduces NSI rates of HCWs.

**Electronic supplementary material:**

The online version of this article (doi:10.1186/s12913-016-1705-y) contains supplementary material, which is available to authorized users.

## Background

Healthcare workers (HCWs) worldwide face the serious occupational health hazard of sharps injuries, commonly referred to as Needle-Stick Injuries (NSIs) [1]. According to the World Health Organization (WHO), there are approximately two million occupational exposures to blood-borne pathogens per year out of the total 35 million estimated HCWs worldwide [2]. In the United States, it was estimated that about 384,325 NSIs occur annually in hospital-based health-care personnel [3]. These injuries account for about one third of all occupational accidents encountered by HCWs in a healthcare setting [4].

Phlebotomy (blood-drawing) procedures alone account for 13 to 62 % of the injuries reported to the Hospital Occupational Health Services [5, 6]. The annual incidence rate of injuries amongst phlebotomists is about 407 per 1000 HCWs, with these blood-drawing procedures accounting for an estimate of 13.3 % of total reported injuries [7]. Similarly, intravenous-access procedures such as the administration of parenteral injections and infusion therapies account for 15.7 % of all reported injuries [7]. In short, blood-involving procedures have higher risks of transferring blood-borne infections than other procedures [8, 9].

Needle-stick injuries are hazardous due to their potential for transmission of blood-borne pathogens, particularly hepatitis B virus (HBV), hepatitis C virus (HCV), and human immunodeficiency virus (HIV) [10, 11]. Indeed, about 40 % of each of HBV and HCV reported cases and about 4.4 % of HIV acquisitions in HCWs are attributable to NSIs [2]. The risk of transmission following a percutaneous injury is 35 % for HBV, 3 to 10 % for HCV, and 0.2 to 0.5 % for HIV [12]. A study published in 2005 reported that up to that date, approximately 66,000, 16,000, and 1000 global HCWs got infected with HBV, HCV, and HIV respectively, due to sharps injuries alone [13].

Safety needle devices possess built-in safety controls that reduce and potentially prevent NSIs [14]. These devices allow needle-safe IV insertion and delivery, blood collection, and intramuscular, intra-dermal and subcutaneous injections [3, 15–17]. We have already systematically reviewed the evidence for intramuscular, intra-dermal and subcutaneous injections (under review for publication). Although individual studies have found a decrease in the number of percutaneous injuries occurring during phlebotomy procedures [17] and intravenous injections [3, 18] upon the use of safety-engineered devices, no reviews to date have analyzed the efficacy of such devices across studies using meta-analysis techniques.

We conducted this study was to gather the evidence necessary for the development by WHO of a policy guidance on use of safety-engineered devices by healthcare workers to deliver a number of procedures, including intravenous and/or phlebotomy procedures. The objective was to systematically review the evidence about the efficacy and safety of using safety-engineered intravenous devices and safety-engineered phlebotomy devices by HCWs in reducing NSIs and/or infection transmission rates.

We opted to review this evidence separately from that of intramuscular and subcutaneous devices whose review we published a while ago [19], for a number of reasons. First, the two types of devices are not interchangeable, so it reasonable to expect that their effects might not be the same. Second, intravenous and phlebotomy devices are associated with a higher rate of infection, compared with intramuscular and subcutaneous devices, given they come in direct contact with blood [20, 21]. Third, very few studies have assessed the two types of devices together. Even those that did, presented the data for the two types of devices separately. Finally, the WHO that commissioned this work was only interested in the evidence for intramuscular and subcutaneous devices.

The specific questions were:

1. What is the efficacy of safety-engineered intravenous devices versus conventional intravenous devices in preventing accidental needle stick injuries when used by HCWs in a health-care setting to perform infusion therapies, and/or intravenous drug administration?
2. What is the efficacy of safety-engineered phlebotomy (blood-withdrawal) devices versus conventional phlebotomy devices in preventing accidental needle stick injuries when used by HCWs in a health-care setting to withdraw blood from patients?

## Methods

While we did not develop a protocol specifically for this systematic review, we based our work on a protocol previously developed for a systematic review on sharp injury prevention syringes for intramuscular, subcutaneous, and intradermal injections [22]. This study adhered to the PRISMA guidelines [23].

### Eligibility criteria

#### Types of studies included

Randomized and non-randomized trial studies, including cohort studies, case-control studies, before and after, and time-series analyses. We excluded abstracts of scientific meetings and conferences, research letters, qualitative studies, letters to the editor, reviews, case reports, and case series.

#### Types of participants and settings

HCWs delivering intravenous therapies and infusions, or drawing blood (venipuncture). We excluded all studies addressing HCWs in non-healthcare settings (e.g. dental clinics, drawing blood at home, or home-based IV therapies). We excluded studies of HCWs delivering intramuscular, intradermal, subcutaneous, articular, intra-cardiac, and intra-peritoneal injections. We excluded studies of blood drawing through capillary sampling (i.e. using lancets).

#### Types of interventions

Introduction into the healthcare setting of a safety device to replace conventional intravenous and/or phlebotomy devices. We included passive but not active devices given the evidence showing the higher efficacy of the former over the latter in reducing NSI [24]. Unlike active devices, passive devices activates automatically during device use and therefore do not require additional steps to initiate the safety mechanism. The intervention could have been accompanied by training of HCWs on how to use the safety-engineered devices and/or by a surveillance system to monitor implementation of the new devices.

Examples of eligible intravenous-related safety devices include: needle-free (or “needle-less”) IV systems, Luer-activated IV administration systems, safe IV catheters with blunt cannula replacing sharp needle, blunt implantable port needles and Needle-less adaptors, and guarded arterio-venous fistula needles. Examples of eligible phlebotomy-related safety devices include: blunt-fill cannulae, vacuum-tube blood collection devices, safety winged butterfly steel needles, and self-retracting and/or self-sheathing (recapping) blood syringes. Ineligible devices include intramuscular, intradermal, subcutaneous, articular, intra-cardiac, and intra-peritoneal needles/syringes. We included studies assessing the introduction of both eligible and ineligible devices, as long as they reported data for eligible devices separately. Additionally, we included studies in which both safety-engineered intravenous devices and safety-engineered phlebotomy devices were introduced as long as they reported separate data for these two types of devices.

#### Types of comparisons

Traditional/conventional non-safety device, such as the ‘single use disposable syringes’, as the comparators.

#### Outcomes of interest

NSI injuries among HCWs as well as HBV, HCV, and/or HIV infections following a NSI among HCWs.

### Literature search

We used the OVID interface to electronically search MEDLINE, EMBASE, CINHAL, and Cochrane Central Register of Controlled Trials (CENTRAL). The searches covered the period beginning with the database inception date and October 2013. We used no language or date restrictions. The Appendix lists the search strategies employed for each database. In addition, we reviewed the references lists of relevant papers, searched personal files for both published and unpublished studies, and contacted experts in the field.

### Selection process

Four reviewers participated in calibration exercises to clarify the eligibility criteria. Then, they screened the titles and abstracts of identified citations for potential eligibility in duplicate (i.e., each citation was screened by two reviewers) and independently using the above described eligibility criteria. We retrieved the full text for any citation judged as potentially eligible by at least one reviewer. Then, the reviewers screened the full texts for eligibility, in duplicate (i.e., each full text was screened by two reviewers) and independently. They used a standardized, pilot-tested full-text screening form. They compared their results and resolved any disagreements through discussion, or with the help of a third reviewer.

### Data extraction and management process

Reviewers extracted data from the eligible studies in a duplicate and independent fashion using a pilot-tested and standardized data-extraction form. They resolved disagreements by discussion or with the help of a third reviewer. For non-English papers, we obtained the translation via Google Translate®.

We extracted the following information from each eligible study: the specific attributes and mode of action of the safety-engineered device; the study design; the characteristics of the participants and of the setting; the intervention employed; the control; the outcomes assessed; the funding source; and disclosures of potential conflicts of interest.

### Risk of bias assessment in the included studies

Reviewers assessed the risk of bias in each included randomized controlled trials in duplicate and independently using the Cochrane risk of bias tool. They resolved all disagreements by discussion or with the help of a third reviewer. The tool includes an assessment of the following criteria for randomized studies: inadequate sequence generation; inadequate allocation concealment; lack of blinding of participants, providers, data collectors, outcome adjudicators, and data analysts; incompleteness of outcome data; selective outcome reporting; and other bias. As for the non-randomized studies, we used the following criteria for assessing the risk of bias: failure to develop and apply appropriate eligibility criteria; flaws in the measurement of exposure/intervention; flaws in the measurement of the outcome(s) of interest; failure to adequately account for, and control for confounding; and incomplete follow-up [25]. We judged each potential source of bias as “high”, “low”, or “unclear”.

### Data synthesis

For categorical data, we calculated for each study the risk ratio (RR) then pooled the results across studies using a random-effects model. We evaluated heterogeneity across studies using the I^2^ test, and considered it to be present when I^2^ is greater than 50 %. Finally, we planned to create inverted funnel plots in order to check for possible publication bias.

### Sensitivity analysis

We identified one study that assessed devices for intravenous and/or phlebotomy procedures, in addition to intramuscular, subcutaneous, and/or intradermal injection procedures, without providing outcome data separately for the different procedures [26]. In a post-hoc decision, we included these studies in the main analysis, but excluded them from a sensitivity analysis, in order to determine their impact on the final results.

### Subgroup analysis

In order to explain any identified heterogeneity, we planned to conduct subgroup analyses based on the following factors: type of procedure for which the device was intended (intravenous or phlebotomy), the type of the device itself, the level of expertise and skills of HCWs, and the time of the injury (before, during, or after the procedure).

### Quality of evidence assessment

We assessed the quality of evidence by outcome, using the GRADE methodology [27]. We then generated a GRADE Evidence Profile to summarize the statistical findings and the quality of evidence obtained for each outcome.

## Results

### Study selection

**Fig. 1:**
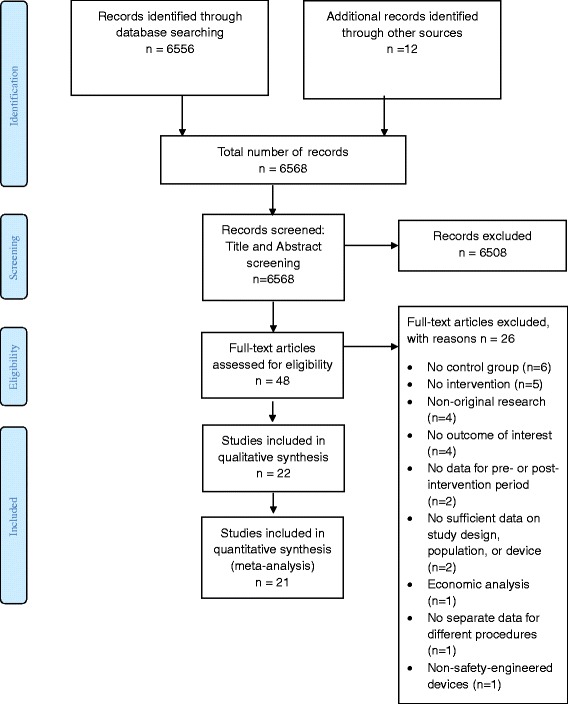
PRISMA flow diagram

Figure 1 shows the study flow diagram. Out of a total of 6566 identified citations, we assessed 46 full texts for eligibility. Of these, we included 22 studies, and excluded the remaining 26 for the following reasons (see Additional file 1: Table S1): lacking a control or standard (conventional device) to which the exposure (safety-engineered device) data can be compared to (*n* = 6) [28–33], not involving any actual intervention and being more of a commentary type of study or using simple observation (*n* = 5) [34–38], being a review, an abstract, or a non-original research paper reporting data from another paper (e.g., magazine articles and monthly issues of hospital reports) (*n* = 4) [39–42], not reporting any data on NSI rates but rather HCWs’ evaluation of the implemented device or its practicality (*n* = 4) [43–46], lacking data for the pre- or the post- intervention period (*n* = 2) [47, 48], lacking sufficient data on study design, population, and device implemented (*n* = 2) [31, 49], reporting economic analysis and cost-relevant data (*n* = 1) [50], not reporting separate data for different procedures (e.g., reporting overall drop in NSI rates where more than one safety device was implemented for different procedures) (*n* = 1) [51], and having the implemented devices as non-safety-engineered devices (*n* = 1) [52]. One of the studies was reported in two peer-reviewed papers (duplicate publication) [5, 6].

### Study characteristics

Additional file 2: Table S2 provides a listing of the twenty-two included studies with detailed description of their characteristics. We summarized these characteristics in the subsequent sections.

### Types of injection

Out of the 22 included studies, twelve studies assessed the introduction of IV safety devices [53–64], five studies assessed the introduction of phlebotomy safety devices [5, 26, 65–67], and five studies assessed the simultaneous introduction of IV and phlebotomy safety devices [68–72]. Four of the studies of the “IV safety devices” category also reported data for subcutaneous, intramuscular, and/or intradermal injection devices separately [68, 70–72]. Five studies of the “phlebotomy safety devices” category also reported data for subcutaneous, intramuscular, and/or intradermal injection devices [26, 68, 70–72], with one of these studies [26] not reporting data separately.

### Brands of devices

Sixteen out of the 22 studies specified the brand name and/or the manufacturing company of the implemented device(s): VanishPoint™ by Retractable Technologies, Inc [72], SafetyGlide™ needles, SafetyGlide TNT insulin units and blunt fill cannulae by Becton-Dickinson [26], Eclipse™, Saf-T E-Z Set™,Preserts™, and Insyte Autoguard™ by Becton Dickinson [71], Surshield™and Versatus-S™ by Terumo [71], Provent Plus™ and Protective Plus™ by Smiths Medical [71], Safety-Lok™ by Becton Dickinson [6, 59, 67], Saf-T Clik™ by Ryan Medical, Inc [65], Clearlink System™ by Baxter Healthcare Corp [53]., Interlink System™ by Baxter Healthcare Corp [54, 56–60, 63]., MasterGuard Anti-Stick Needle Protector™ by Medisystems Corp [66]., Safesite System™ by Braun Medical, Inc [64], Lifeshield™ by Abbott Laboratories [56], Saf-Site™ by Burron Medical [56], Punctur-Guard™ by Bio-Plexus, Inc [6], and Venipuncture Needle-Pro™ by Smiths Medical [6]. The remaining six studies mentioned neither the brand name of the device nor the manufacturer or supplier.

### Funding

Nine out of the 22 studies reported their funding sources; these include:

1. Becton Dickinson; [26]
2. National Institute of Allergy and Infectious Diseases; the Centers for Disease Control and Prevention; and the Prevention Epicenters; [70]
3. The Directorate General of Public Health of the Autonomous Community of Valencia, Spain; [71]
4. The Educational Resource Centers, Inc, at the National Institute for Occupational Safety and Health; [57]
5. New York State Department of Health and Braun Medical, Inc. [64]
6. The Centers for Disease Control and Prevention/National Institute of Occupational Safety and Health; Mr. William E. Flanagan Jr; [55]
7. The French Ministry of Health and la Mutuelle Nationale des Hospitaliers and the following companies: Becton-Dickinson, Bristol-Myers-Squibb, Glaxo Wellcome, Johnson & Johnson Medical, Kendall Sherwood David & Geck, MAPA Hutchinson, Merck Sharp & Dohme Chibret, Sanofi Winthrop, SIMS France, and Terumo; [69]
8. Baxter Healthcare Corporation [53, 59].

### Disclosure of conflicts of interest

Only two studies had their authors declare in writing at the end that they have no conflicts of interest [68, 71]. The remaining 20 studies did not report any disclosures about potential conflicts of interest.

### Study design

Only one study was a randomized controlled trial [56] and assessed the introduction of IV safety devices with a prospective data collection design. The remaining 21 studies were non-randomized employing a before-and-after study design. Of these 21 studies, four collected data retrospectively throughout the study period [53, 57, 62, 68], three collected data retrospectively for the “before” period and prospectively for the “after” period [54, 64, 66], eight collected data prospectively throughout the study period [26, 55, 59, 67, 69–72], and one collected data retrospectively for the “before period” but was unclear with regards to the “after” period [65]. The five remaining non-randomized studies did not specify their data collection approach [6, 58, 60, 61, 63].

### Participants

The included studies involved hospital-based nursing staff (*n* = 20) [6, 26, 53–64, 67–72], clinical staff of hospital-affiliated hemodialysis units (*n* = 1) [66] laboratory personnel and non-nursing phlebotomists (*n* = 7) [6, 55, 57, 62, 65, 68, 70], ancillary/outpatient staff (*n* = 3) [26, 55, 70], housekeeping staff (*n* = 7) [53, 57, 58, 62, 70–72], hospital aide (*n* = 1) [58], surgical and operation room staff (*n* = 7) [26, 53, 58, 59, 62, 68, 70], ambulatory care HCWs (*n* = 1) [60], attending physicians (*n* = 9) [26, 53, 58, 59, 62, 68, 70–72], interns, residents, and fellows (*n* = 6) [6, 26, 57, 59, 70, 71], medical students (*n* = 3) [6, 59, 68], and nursing students (*n* = 2) [59, 71].

### Settings

The included studies were all conducted in high-income countries, as follows: United States (*n* = 13) [6, 54–57, 59–62, 64–66, 70], Canada (*n* = 2) [53, 63], France (*n* = 2) [67, 69], United Kingdom (*n* = 1) [26], Germany (*n* = 1) [68], Spain (*n* = 1) [71], Australia (*n* = 1) [72], and New Zealand (*n* = 1) [58].

### Intervention/exposure

Interventions involved the introduction of a variety of IV and/or phlebotomy safety devices, specified above under “Device brand”, into a healthcare setting. In 17 out of the 22 included studies, the HCWs received some form of educational intervention and/or training on the use of the newly implemented device [26, 54–57, 59–64, 66–68, 70–72].

### Control/comparison

Nineteen out of the 22 included studies mentioned that the standard of comparison to which the exposure was compared, was the use of “conventional”, “standard”, “traditional”, or “alternative” devices for the corresponding IV and/or Phlebotomy procedure(s) under study [6, 53–56, 59–71] Only three studies did not specify the nature of the device used as control [57, 58, 72]. Additionally, one of the studies used “standard education” and “enhanced training” in both the control and exposure groups to make the availability of the safety device(s) under study the only variable between the two groups [26].

### Outcomes

All of the included studies assessed NSI among HCWs with and without the introduction of a safety device. Only one of the included studies reported data narratively on patient infection(s) with blood-borne pathogens (HBV, HCV, and HIV) upon acquiring NSIs [68]. All other studies reported no valuable data on any of the outcomes of interest other than NSIs.

### Risk of bias within studies

**Fig. 2:**
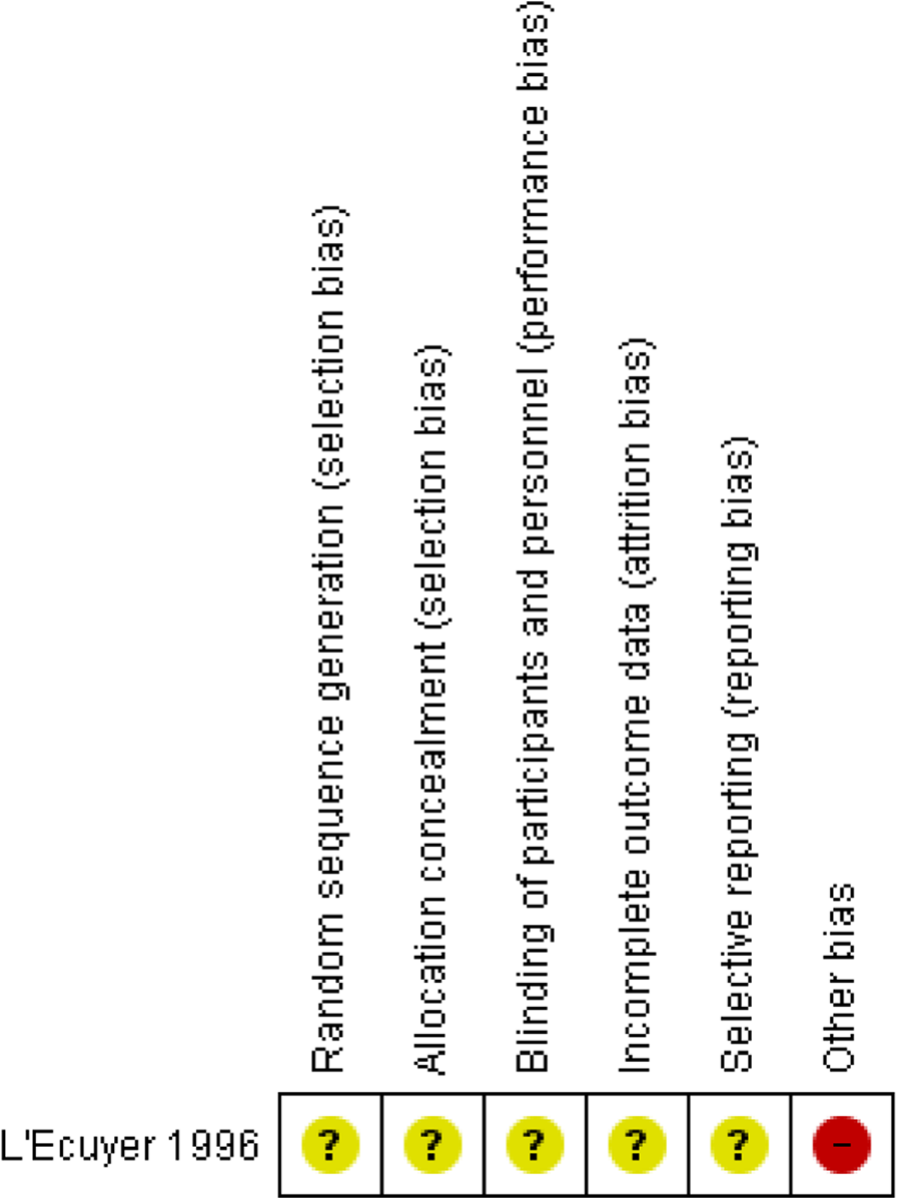
Risk of bias summary diagram for the single included randomized study assessing IV safety devices

**Fig. 3:**
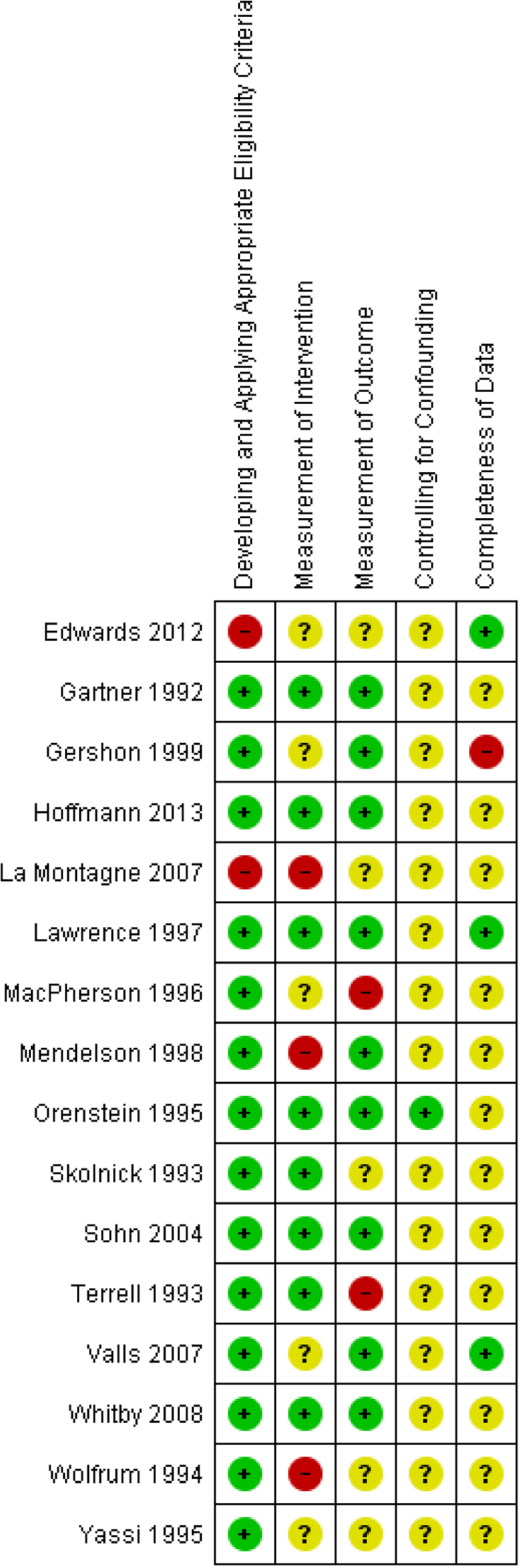
Risk of bias summary diagram for all the included non-randomized studies assessing IV safety devices

**Fig. 4:**
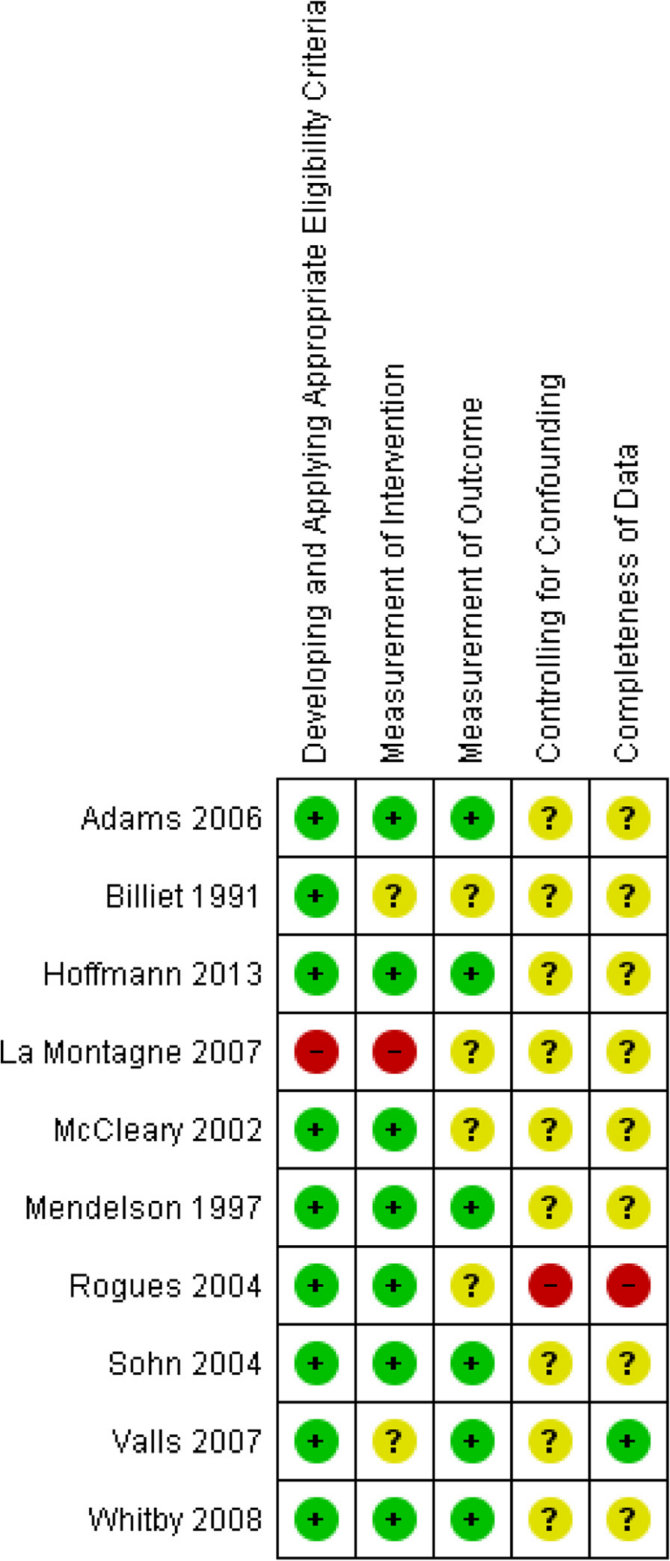
Risk of bias summary diagram for all the included non-randomized studies assessing Phlebotomy (blood-drawing) safety devices

Additional files 3 and 4: Table S3 and S4 detail the risk of bias assessment and the underlying judgments for the included randomized study [56], and the remaining non-randomized studies respectively. These assessments are summarized graphically in Fig. 2 (the randomized study), Fig. 3 (non-randomized studies of intravenous devices) and Fig. 4 (non-randomized studies of phlebotomy devices).

### Meta-analysis for intravenous safety devices

The eligible studies that reported NSI data used three main types of statistics: incidence of NSI per HCW (*n* = 5) [55, 57, 68, 70, 72], incidence of NSI per devices used or procedures performed (*n* = 4) [54, 58, 69, 71], and incidence of NSI per year (*n* = 6) [53, 59–61, 63, 64]. The randomized study reported the incidence of NSIs per patient-days [56]. We performed distinct meta-analyses for these different statistics.

We did not include one of the studies in any meta-analysis because it did not report the statistical data in any of the three types of statistics mentioned above [62]. That study reported a 39 % decrease of needle-stick injuries over a 4-year period following the introduction of a safety-designed needle-free IV access system.

#### NSI per HCW

**Fig. 5:**
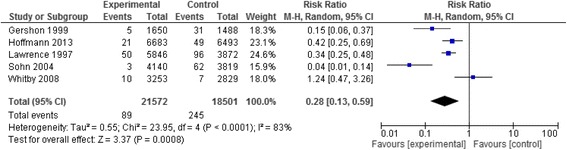
Needle stick injury data of the studies assessing IV safety devices reported as rates of injuries per number of healthcare workers

The meta-analysis of four studies [55, 57, 68, 70, 72] resulted in a pooled relative risk of 0.28 [95 % CI 0.13, 0.59]. The I^2^ value was 83 % (Fig. 5). We were not concerned about heterogeneity given most studies showed benefit and the heterogeneity reflected the variation in the degree of that benefit. We rated up the quality of evidence from low (observational data) to moderate due to the large effect size, while acknowledging some concern about risk of bias in the included studies.

#### NSI per device or procedure performed

**Fig. 6:**
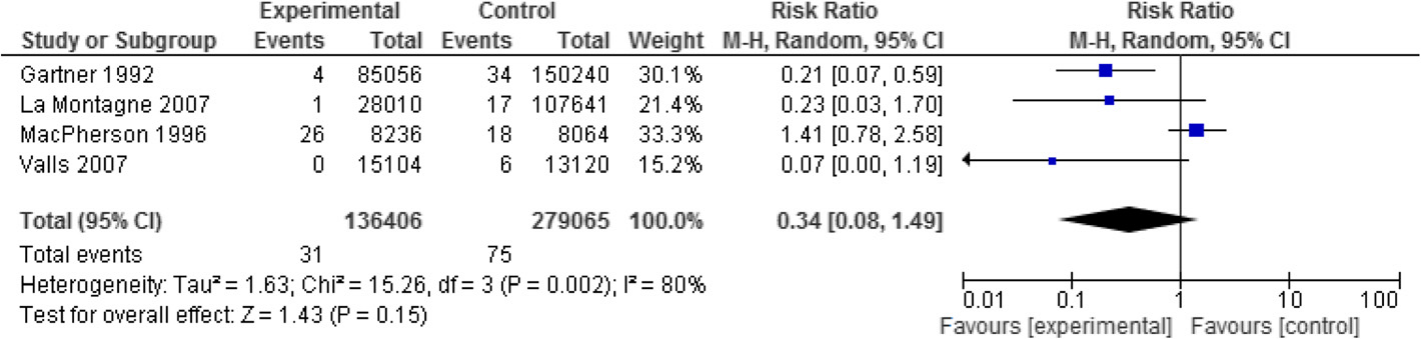
Needle stick injury data of the studies assessing IV safety devices reported as rates of injuries per number of devices or procedures performed

The meta-analysis of four studies [54, 58, 69, 71] resulted in a pooled relative risk of 0.34 [95 % Confidence Interval (CI) 0.08,1.49]. The I^2^ value was 80 % (Fig. 6). We were not concerned about heterogeneity given most studies showed benefit and the heterogeneity reflected the variation in the degree of that benefit. While the large observed effect would typically warrant rating up the quality of evidence from low to moderate, we did not do so because of the imprecision of the results. We judged the quality of evidence as low.

#### NSI per year

**Fig. 7:**
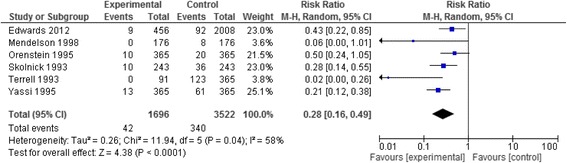
Needle stick injury data of the studies assessing IV safety devices reported as rates of injuries per year

The meta-analysis of six studies [53, 59–61, 63, 64] resulted in a pooled relative risk of 0.28 [95 % CI 0.16, 0.49]. The I^2^ value was 58 % (Fig. 7). We were not concerned about heterogeneity given most studies showed benefit and the heterogeneity reflected the variation in the degree of that benefit. We rated up the quality of evidence from low (observational data) to moderate due to the large effect size, while acknowledging some concern about risk of bias in the included studies.

**Fig. 8:**
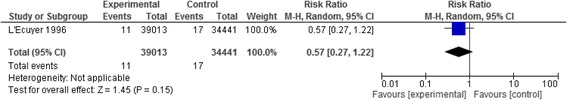
Needle stick injury data of the randomized trial assessing IV safety devices, reported as rates of injuries per patient-days

In a separate analysis, we included the only randomized controlled trial [56] whose relative risk ratio turned out to be 0.57 [95 % CI 0.27, 1.22] (Fig. 8). We rated the quality of evidence as low due to concern about risk of bias and imprecision.

### Meta-analysis for phlebotomy safety devices

The eligible studies that reported NSI data used two main types of statistics: incidence of NSI per HCW (*n* = 3) [68, 70, 72], and incidence of NSI per devices used or procedures performed (*n* = 7) [6, 26, 65–67, 69, 71]. We performed distinct meta-analyses for these different statistics. We excluded only one study from the meta-analysis because it had an interrupted-time series design (ITS) [67]. That study reported an overall reduction of 48 % in percutaneous injuries per 100,000 phlebotomies performed.

#### NSI per HCW

**Fig. 9:**
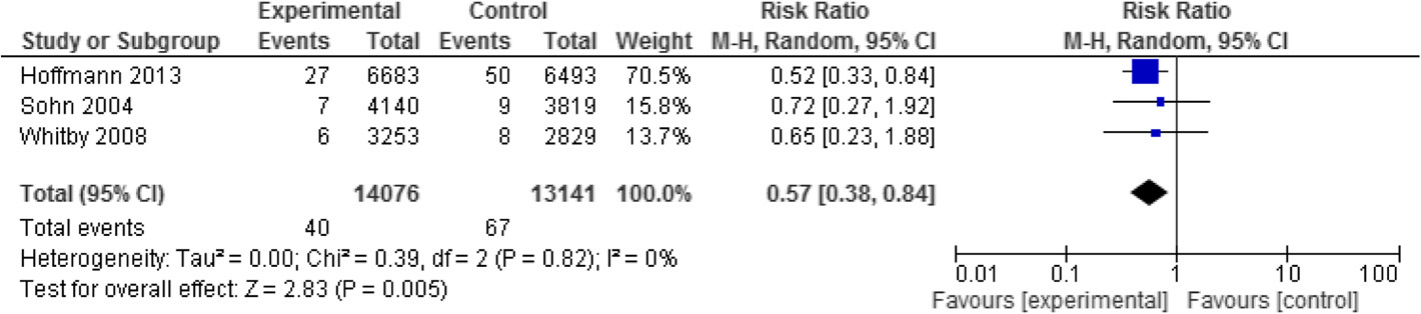
Needle stick injury data of the studies assessing phlebotomy safety devices reported as rates of injuries per number of healthcare workers

The meta-analysis of two studies [68, 70, 72] resulted in a pooled relative risk of 0.57 [95 % CI 0.38, 0.84]. The I^2^ value was 0 % (Fig. 9). We rated up the quality of evidence from low (observational data) to moderate due to the large effect size.

#### NSI per device or procedure performed

**Fig. 10:**
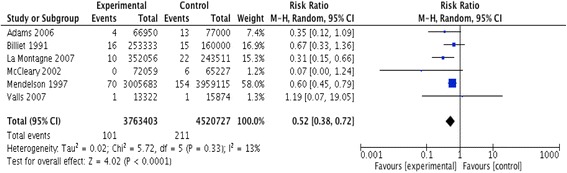
Needle stick injury data of the studies assessing phlebotomy safety devices reported as rates of injuries per number of devices or procedures performed

The meta-analysis of six studies [6, 26, 65, 66, 69, 71] resulted in a pooled relative risk of 0.52 [95 % CI 0.38, 0.72]. The I^2^ value was 13 % (Fig. 10). We rated up the quality of evidence from low (observational data) to moderate due to the large effect size.

### Other outcomes

One study [68] reported blood-borne pathogen infection rates in patients, and not HCWs, for the year before introducing the safety devices and in the year afterwards. It stated: “Infection with HBV, HCV or HIV in needle-stick index patients was high in both years, with a prevalence of 6.7 % in 2007 and 9.0 % in 2009 for all three blood-borne viruses together”. No information about statistical significance was provided. Another study [72] stated: “No significant increase in bloodstream infections was detected during the study period”, without any numerical evidence included to support that statement. None of the other studies reported similar data relevant to these other outcomes of interest such as reduction in HBV, HCV, and HIV infections among HCWs and/or patients, or reduction in any other blood-borne infection in HCWs and/or patients.

### Additional analyses

The post-hoc sensitivity analysis excluding the one study not providing outcome data separately for the different types of procedures [26], did not substantively impact the results of the main analysis. We were not able to conduct planned subgroup analyses because of the relatively small number of studies per analysis. Another reason was the lack of sufficient and clearly reported data on some of the factors we planned to conduct the analyses based upon such as: the type of device, the level of expertise of HCWs using the devices, the time of injury (before, during, or after the injection/withdrawal), and the mechanism of action/use of the specific device under study.

## Discussion

In summary, we identified moderate quality evidence that intravenous safety devices and phlebotomy safety devices reduce the risk of NSIs amongst HCWs performing such procedures. We did not identify substantial evidence about the effects on HCWs infection(s) with blood-borne pathogens (HBV, HCV, and HIV).

We have identified a systematic review published in 2006 addressing the same question [73]. That review concluded that “a reduction in injury rate of ~50–60 % might be possible with phlebotomy devices”, which is less than the current review’s estimates. We believe our findings are more reliable for a number of reasons. First, that review addressed the efficacy of safety devices with either active or passive safety features. Our review focused on passive devices given there is evidence showing their higher efficacy compared with active devices [24]. Additional advantages of our review include the use of a systematic approach to study selection and data abstraction, the assessment of risk of bias of included studies, and the grading of the quality of evidence by outcome using the GRADE methodology. The reviewers included seven studies in common with our review [56, 57, 59, 63, 65, 67, 70]. They also included four studies that we excluded for different reasons: the lack of an actual implementation of a safety device [38], not reporting data separately for separate procedures [51], being an abstract and not a published full-text [42], and not reporting comparative data for the conventional device [30]. Thus, compared with the 2006 review, our systematic review included 15 additional studies [6, 26, 53–55, 58, 60–62, 64, 66, 68, 69, 71, 72].

We have identified a more recently published Cochrane systematic review addressing a related but distinct research question [74]. In fact, that review had a wider scope and addressed types of safety devices other than intravenous and/or phlebotomy (e.g., sharps containers for on-spot disposal of used sharps) and in settings other than hospitals (e.g., dental clinics). Also, the Cochrane review included studies of safety-devices with active features, which we excluded as justified above under ‘Types of interventions’. Unlike our review, the Cochrane review excluded before and after studies.

The Cochrane review found very low quality evidence that safety-engineered blood-collection (i.e., phlebotomy) and IV devices can lower NSIs compared to the conventional non-safety-engineered devices. However, one of their two estimates of relative risk based on one trial was higher than ours (0.62 (95 % CI 0.27 to 1.41) while their other estimate of relative risk based on one controlled before and after study was lower than ours (0.06 (95 % CI 0.0 to 1.09). This discrepancy between the two reviews is due to the different eligibility criteria, particularly in terms of our inclusion of six non-controlled before and after studies [53–55, 57, 61, 72].

There are two main explanations for the differences in rating the quality of evidence between the Cochrane review and our review. First, the inter-rater reliability of using the GRADE approach for assessing the quality of evidence is not perfect, with an inter-rater reliability (IRR) of 0.72 among members of the GRADE working group. Second, there is a debate on how to rate the quality of evidence from observational studies, with some advocating rating evidence from studies using ITS analysis as moderate quality [75]. Our approach was to start with low quality rating for the evidence from uncontrolled before and after studies, then rating them up for large effect [76].

Few studies have assessed the use of these devices from the economic point of view. Griswold et al. [50] assessed StatLock™, a safety-engineered device designed to protect HCWs placing central venous catheters from NSIs. The authors estimated that the use of the device could spare hospitals a cost of $2723 incurred by each NSI acquired by a HCW, and could have saved a minimum of $57,183 over the evaluated four-year study period. Yassi et al. conducted economic-benefit and cost effectiveness analyses for the use of safety devices in intravenous and phlebotomy procedures [63]. They found that the introduction of the safety Interlink system may increase cost to the hospital, but judged that any incremental cost would be offset by the avoidance of costs related to NSIs and infections. One study evaluated the ease of use of the safety devices. Griswold et al. found that surgical residents “seemed to prefer using sutures over the StatLock device” [50]. One participant suggested the need for additional practice with the device before using it in a clinical setting.

The main strength of this review is the use of a rigorous methodology for conducting systematic reviews. The major limitation pertained to the lack of original studies assessing the effects of the safety devices on blood borne infections (particularly HIV, HBV, and HCV) amongst workers healthcare. Also the quality of evidence for some of the outcomes of interest was low, suggesting the need for further studies to strengthen that quality.

## Conclusion

The findings of this review have significant implications for HCWs. The introduction into the healthcare setting of safety-engineered devices for intravenous and/or phlebotomy procedures will likely reduce NSIs to HCWs. However, the decision to introduce these devices into healthcare facilities should take into account the costs related to the purchase, training, use of these devices and the impact of their introduction on the sharps waste generated and its safe management. These devices are typically introduced as part of a wider injection safety program, including: education about the risks associated with accidental injuries, training in the safety devices, surveillance and reporting of NSIs, immunization against HBV, post exposure prophylaxis and appropriate sharps waste management. Also, the HCWs would need to be involved in evaluating and selecting the proper devices.

The findings also have important implications for future research. There is a need to further build the evidence base for safety-engineered devices for intravenous and/or phlebotomy procedures. There is also a need for studies assessing the economic impact of these devices, the ease of their use in practice, as well as their acceptability by HCWs. Finally, all included studies were conducted in high income countries; there is a need to undertake such studies in middle- and low- income countries where unsafe injection practices and accidental needle stick injuries in HCWs are still prevalent.

## Appendix

Detailed description of the search strategy used with the MeSH terms and keywords employed.

Search strategies used to detect relevant papers

Search strategy used in Medline:

1. Health Personnel/
2. Personnel, Hospital/
3. ((Healthcare or health-care or (health adj care)) adj2 worker*).mp.
4. Paramedic*.mp.
5. ((medical or nurs*or ancillary) adj2 staff*).mp.
6. (Medical adj2 laboratory adj2 techn*).mp.
7. Pharmacist*.mp.
8. physician*.mp.
9. Hospitalist*.mp.
10. internist*.mp.
11. doctor*.mp.
12. Phlebotomist*.mp.
13. exp Needlestick Injuries/
14. exp Accidents, Occupational/and (syringe* or needle* or inject*).mp.
15. (injur* adj3 (syringe* or needle* or inject*)).mp.
16. exp Accidents, Occupational/and (syringe* or needle* or inject*).mp.
17. exp Accident Prevention/and (syringe* or needle* or inject*).mp.
18. (blood adj3 collection adj3 (syringe* or needle* or system* or device* or material* or product* or set*)).mp.
19. ((need-less or needless or needle-free or needlefree) adj3 (syringe* or needle* or system* or device* or material* or product* or set* or inject*)).mp.
20. (Single adj3 “use” adj3 (syringe* or needle* or system* or device* or material* or product* or set* or inject*)).mp.
21. (prevent* adj3 (syringe* or needle* or system* or device* or material* or product* or set* or inject*)).mp.
22. (reuse adj3 (syringe* or needle* or system* or device* or material* or product* or set* or inject*)).mp.
23. (exp Equipment Reuse/or exp Disposable equipment/) and (syringe* or needle* or system* or device* or material* or product* or set* or inject*).mp.
24. (Disposable adj2 equipment* adj3 (syringe* or needle* or inject*)).mp.
25. ((prefill* or pre-fill*) adj3 (syringe* or needle* or inject*)).mp.
26. (Autopen or auto-pen).mp.
27. “Vetter Lyo-ject”.mp.
28. Vasceze.mp.
29. Sterimatic.mp.
30. “Safe-Point”.mp.
31. “Needle-Pro”.mp.
32. Hypak.mp.
33. VanishPoint.mp.
34. “Slip-lock”.mp.
35. Luerlok.mp.
36. “Bio-Set”.mp.
37. “Auto-disposable syringe*”.mp.
38. ((prefill* or pre-fill*) adj2 syringe*).mp.
39. “BD Hypak”.mp.
40. “Safety-Lok”.mp.
41. (Kendall’s adj2 Monoject).mp.
42. “autodestruct syringe”.mp.
43. SoloShot.mp.
44. “Monodose syringe*”.mp.
45. “Unifine pentip*”.mp.
46. Autoject.mp.
47. (ultrasafe adj passive adj delivery adj system).mp.
48. “Tip-Lok”.mp.
49. “Gettig Guard”.mp.
50. “Inviro SNAP!”.mp.
51. “Maxxon safety syringe*”.mp.
52. “monoject magellan”.mp.
53. “needle-pro”.mp.
54. “point-lok”.mp.
55. “wandplus”.mp.
56. “safetyglide”.mp.
57. “safety wand”.mp.
58. “powder ject”.mp.
59. or/1–12
60. or/13–58
61. and/59–60

Search strategy used in EMBASE:

1. Health Personnel/
2. Personnel, Hospital/
3. ((Healthcare or health-care or (health adj care)) adj2 worker*).mp. [mp = title, abstract, subject headings, heading word, drug trade name, original title, device manufacturer, drug manufacturer, device trade name, keyword]
4. Paramedic*.mp. [mp = title, abstract, subject headings, heading word, drug trade name, original title, device manufacturer, drug manufacturer, device trade name, keyword]
5. ((medical or nurs*or ancillary) adj2 staff*).mp. [mp = title, abstract, subject headings, heading word, drug trade name, original title, device manufacturer, drug manufacturer, device trade name, keyword]
6. (Medical adj2 laboratory adj2 techn*).mp. [mp = title, abstract, subject headings, heading word, drug trade name, original title, device manufacturer, drug manufacturer, device trade name, keyword]
7. Pharmacist*.mp. [mp = title, abstract, subject headings, heading word, drug trade name, original title, device manufacturer, drug manufacturer, device trade name, keyword]
8. physician*.mp. [mp = title, abstract, subject headings, heading word, drug trade name, original title, device manufacturer, drug manufacturer, device trade name, keyword]
9. Hospitalist*.mp. [mp = title, abstract, subject headings, heading word, drug trade name, original title, device manufacturer, drug manufacturer, device trade name, keyword]
10. internist*.mp. [mp = title, abstract, subject headings, heading word, drug trade name, original title, device manufacturer, drug manufacturer, device trade name, keyword]
11. doctor*.mp. [mp = title, abstract, subject headings, heading word, drug trade name, original title, device manufacturer, drug manufacturer, device trade name, keyword]
12. Phlebotomist*.mp. [mp = title, abstract, subject headings, heading word, drug trade name, original title, device manufacturer, drug manufacturer, device trade name, keyword]
13. exp Needlestick Injuries/
14. exp Accidents, Occupational/and (syringe* or needle* or inject*).mp. [mp = title, abstract, subject headings, heading word, drug trade name, original title, device manufacturer, drug manufacturer, device trade name, keyword]
15. (injur* adj3 (syringe* or needle* or inject*)).mp. [mp = title, abstract, subject headings, heading word, drug trade name, original title, device manufacturer, drug manufacturer, device trade name, keyword]
16. exp Accidents, Occupational/and (syringe* or needle* or inject*).mp. [mp = title, abstract, subject headings, heading word, drug trade name, original title, device manufacturer, drug manufacturer, device trade name, keyword]
17. exp Accident Prevention/and (syringe* or needle* or inject*).mp. [mp = title, abstract, subject headings, heading word, drug trade name, original title, device manufacturer, drug manufacturer, device trade name, keyword]
18. (blood adj3 collection adj3 (syringe* or needle* or system* or device* or material* or product* or set*)).mp. [mp = title, abstract, subject headings, heading word, drug trade name, original title, device manufacturer, drug manufacturer, device trade name, keyword]
19. ((need-less or needless or needle-free or needlefree) adj3 (syringe* or needle* or system* or device* or material* or product* or set* or inject*)).mp. [mp = title, abstract, subject headings, heading word, drug trade name, original title, device manufacturer, drug manufacturer, device trade name, keyword]
20. (Single adj3 “use” adj3 (syringe* or needle* or system* or device* or material* or product* or set* or inject*)).mp. [mp = title, abstract, subject headings, heading word, drug trade name, original title, device manufacturer, drug manufacturer, device trade name, keyword]
21. (prevent* adj3 (syringe* or needle* or system* or device* or material* or product* or set* or inject*)).mp. [mp = title, abstract, subject headings, heading word, drug trade name, original title, device manufacturer, drug manufacturer, device trade name, keyword]
22. (reuse adj3 (syringe* or needle* or system* or device* or material* or product* or set* or inject*)).mp. [mp = title, abstract, subject headings, heading word, drug trade name, original title, device manufacturer, drug manufacturer, device trade name, keyword]
23. (exp Equipment Reuse/or exp Disposable equipment/) and (syringe* or needle* or system* or device* or material* or product* or set* or inject*).mp. [mp = title, abstract, subject headings, heading word, drug trade name, original title, device manufacturer, drug manufacturer, device trade name, keyword]
24. (Disposable adj2 equipment* adj3 (syringe* or needle* or inject*)).mp. [mp = title, abstract, subject headings, heading word, drug trade name, original title, device manufacturer, drug manufacturer, device trade name, keyword]
25. ((prefill* or pre-fill*) adj3 (syringe* or needle* or inject*)).mp. [mp = title, abstract, subject headings, heading word, drug trade name, original title, device manufacturer, drug manufacturer, device trade name, keyword]
26. (Autopen or auto-pen).mp. [mp = title, abstract, subject headings, heading word, drug trade name, original title, device manufacturer, drug manufacturer, device trade name, keyword]
27. “Vetter Lyo-ject”.mp. [mp = title, abstract, subject headings, heading word, drug trade name, original title, device manufacturer, drug manufacturer, device trade name, keyword]
28. Vasceze.mp. [mp = title, abstract, subject headings, heading word, drug trade name, original title, device manufacturer, drug manufacturer, device trade name, keyword]
29. Sterimatic.mp. [mp = title, abstract, subject headings, heading word, drug trade name, original title, device manufacturer, drug manufacturer, device trade name, keyword]
30. “Safe-Point”.mp. [mp = title, abstract, subject headings, heading word, drug trade name, original title, device manufacturer, drug manufacturer, device trade name, keyword]
31. “Needle-Pro”.mp. [mp = title, abstract, subject headings, heading word, drug trade name, original title, device manufacturer, drug manufacturer, device trade name, keyword]
32. Hypak.mp. [mp = title, abstract, subject headings, heading word, drug trade name, original title, device manufacturer, drug manufacturer, device trade name, keyword]
33. VanishPoint.mp. [mp = title, abstract, subject headings, heading word, drug trade name, original title, device manufacturer, drug manufacturer, device trade name, keyword]
34. “Slip-lock”.mp. [mp = title, abstract, subject headings, heading word, drug trade name, original title, device manufacturer, drug manufacturer, device trade name, keyword]
35. Luerlok.mp. [mp = title, abstract, subject headings, heading word, drug trade name, original title, device manufacturer, drug manufacturer, device trade name, keyword]
36. “Bio-Set”.mp. [mp = title, abstract, subject headings, heading word, drug trade name, original title, device manufacturer, drug manufacturer, device trade name, keyword]
37. “Auto-disposable syringe*”.mp. [mp = title, abstract, subject headings, heading word, drug trade name, original title, device manufacturer, drug manufacturer, device trade name, keyword]
38. ((prefill* or pre-fill*) adj2 syringe*).mp. [mp = title, abstract, subject headings, heading word, drug trade name, original title, device manufacturer, drug manufacturer, device trade name, keyword]
39. “BD Hypak”.mp. [mp = title, abstract, subject headings, heading word, drug trade name, original title, device manufacturer, drug manufacturer, device trade name, keyword]
40. “Safety-Lok”.mp. [mp = title, abstract, subject headings, heading word, drug trade name, original title, device manufacturer, drug manufacturer, device trade name, keyword]
41. (Kendall’s adj2 Monoject).mp. [mp = title, abstract, subject headings, heading word, drug trade name, original title, device manufacturer, drug manufacturer, device trade name, keyword]
42. “autodestruct syringe”.mp. [mp = title, abstract, subject headings, heading word, drug trade name, original title, device manufacturer, drug manufacturer, device trade name, keyword]
43. SoloShot.mp. [mp = title, abstract, subject headings, heading word, drug trade name, original title, device manufacturer, drug manufacturer, device trade name, keyword]
44. “Monodose syringe*”.mp. [mp = title, abstract, subject headings, heading word, drug trade name, original title, device manufacturer, drug manufacturer, device trade name, keyword]
45. “Unifine pentip*”.mp. [mp = title, abstract, subject headings, heading word, drug trade name, original title, device manufacturer, drug manufacturer, device trade name, keyword]
46. Autoject.mp. [mp = title, abstract, subject headings, heading word, drug trade name, original title, device manufacturer, drug manufacturer, device trade name, keyword]
47. (ultrasafe adj passive adj delivery adj system).mp. [mp = title, abstract, subject headings, heading word, drug trade name, original title, device manufacturer, drug manufacturer, device trade name, keyword]
48. “Tip-Lok”.mp. [mp = title, abstract, subject headings, heading word, drug trade name, original title, device manufacturer, drug manufacturer, device trade name, keyword]
49. “Gettig Guard”.mp. [mp = title, abstract, subject headings, heading word, drug trade name, original title, device manufacturer, drug manufacturer, device trade name, keyword]
50. “Inviro SNAP!”.mp. [mp = title, abstract, subject headings, heading word, drug trade name, original title, device manufacturer, drug manufacturer, device trade name, keyword]
51. “Maxxon safety syringe*”.mp. [mp = title, abstract, subject headings, heading word, drug trade name, original title, device manufacturer, drug manufacturer, device trade name, keyword]
52. “monoject magellan”.mp. [mp = title, abstract, subject headings, heading word, drug trade name, original title, device manufacturer, drug manufacturer, device trade name, keyword]
53. “needle-pro”.mp. [mp = title, abstract, subject headings, heading word, drug trade name, original title, device manufacturer, drug manufacturer, device trade name, keyword]
54. “point-lok”.mp. [mp = title, abstract, subject headings, heading word, drug trade name, original title, device manufacturer, drug manufacturer, device trade name, keyword]
55. “wandplus”.mp. [mp = title, abstract, subject headings, heading word, drug trade name, original title, device manufacturer, drug manufacturer, device trade name, keyword]
56. “safetyglide”.mp. [mp = title, abstract, subject headings, heading word, drug trade name, original title, device manufacturer, drug manufacturer, device trade name, keyword]
57. “safety wand”.mp. [mp = title, abstract, subject headings, heading word, drug trade name, original title, device manufacturer, drug manufacturer, device trade name, keyword]
58. “powder ject”.mp. [mp = title, abstract, subject headings, heading word, drug trade name, original title, device manufacturer, drug manufacturer, device trade name, keyword]
59. or/1–12
60. or/13–58
61. and/59–60

Search strategy used in CENTRAL:

1. MeSH descriptor: [Health Personnel] explode all trees
2. MeSH descriptor: [Personnel, Hospital] explode all trees
3. ((Healthcare or health-care or (health near/1 care)) near/2 worker*): ti,ab,kw (Word variations have been searched)
4. Paramedic*: ti,ab,kw (Word variations have been searched)
5. ((medical or nurs*OR ancillary) near/2 staff*):ti,ab,kw (Word variations have been searched)
6. (Medical near/2 laboratory near/2 techn*):ti,ab,kw (Word variations have been searched)
7. Pharmacist*:ti,ab,kw (Word variations have been searched)
8. physician*:ti,ab,kw (Word variations have been searched)
9. Hospitalist*:ti,ab,kw (Word variations have been searched)
10. internist*:ti,ab,kw (Word variations have been searched)
11. doctor*:ti,ab,kw (Word variations have been searched)
12. Phlebotomist*: ti,ab,kw (Word variations have been searched)
13. MeSH descriptor: [Needlestick Injuries] explode all trees
14. MeSH descriptor: [Accidents, Occupational] explode all trees
15. (syringe* or needle* or inject*): ti,ab,kw (Word variations have been searched)
16. #14 and #15
17. (injur* near/3 (syringe* or needle* or inject*)): ti,ab,kw (Word variations have been searched)
18. MeSH descriptor: [Accident Prevention] explode all trees
19. (syringe* or needle* or inject*): ti,ab,kw (Word variations have been searched)
20. #18 and #19
21. (blood near/3 collection near/3 (syringe* or needle* or system* or device* or material* or product* or set*)): ti,ab,kw (Word variations have been searched)
22. ((need-less or needless or needle-free or needlefree) near/3 (syringe* or needle* or system* or device* or material* or product* or set* or inject*)): ti,ab,kw (Word variations have been searched)
23. (Single near/3 “use” near/3 (syringe* or needle* or system* or device* or material* or product* or set* or inject*)):ti,ab,kw (Word variations have been searched)
24. (prevent* near/3 (syringe* or needle* or system* or device* or material* or product* or set* or inject*)):ti,ab,kw (Word variations have been searched)
25. (reuse near/3 (syringe* or needle* or system* or device* or material* or product* or set* or inject*)): ti,ab,kw (Word variations have been searched)
26. MeSH descriptor: [Equipment Reuse] explode all trees
27. MeSH descriptor: [Disposable Equipment] explode all trees
28. #26 or #27
29. (syringe* or needle* or system* or device* or material* or product* or set* or inject*): ti,ab,kw (Word variations have been searched)
30. #28 and #29
31. (Disposable near/2 equipment* near/3 (syringe* or needle* or inject*)): ti,ab,kw (Word variations have been searched)
32. ((prefill* or pre-fill*) near/3 (syringe* or needle* or inject*)): ti,ab,kw (Word variations have been searched)
33. ((prefill* or pre-fill*) near/3 (syringe* or needle* or inject*)): ti,ab,kw (Word variations have been searched)
34. (Autopen or auto-pen): ti,ab,kw (Word variations have been searched)
35. “Vetter Lyo-ject”: ti,ab,kw (Word variations have been searched)
36. Vasceze: ti,ab,kw (Word variations have been searched)
37. Sterimatic: ti,ab,kw (Word variations have been searched)
38. “Safe-Point”: ti,ab,kw (Word variations have been searched)
39. “Needle-Pro”: ti,ab,kw (Word variations have been searched)
40. Hypak:ti,ab,kw (Word variations have been searched)
41. VanishPoint: ti,ab,kw (Word variations have been searched)
42. Slip-lock: ti,ab,kw (Word variations have been searched)
43. Luerlok: ti,ab,kw (Word variations have been searched)
44. “Bio-Set”: ti,ab,kw (Word variations have been searched)
45. “Auto-disposable syringe*”: ti,ab,kw (Word variations have been searched)
46. ((prefill* or pre-fill*) near/3 syringe*): ti,ab,kw (Word variations have been searched)
47. “BD Hypak”: ti,ab,kw (Word variations have been searched)
48. “Safety-Lok”: ti,ab,kw (Word variations have been searched)
49. (Kendall’s adj2 Monoject): ti,ab,kw (Word variations have been searched)
50. “autodestruct syringe”: ti,ab,kw (Word variations have been searched)
51. SoloShot: ti,ab,kw (Word variations have been searched)
52. “Monodose syringe*”: ti,ab,kw (Word variations have been searched)
53. “Unifine pentip*”: ti,ab,kw (Word variations have been searched)
54. Autoject: ti,ab,kw (Word variations have been searched)
55. (ultrasafe near passive near delivery near system): ti,ab,kw (Word variations have been searched)
56. “Tip-Lok”: ti,ab,kw (Word variations have been searched)
57. “Gettig Guard”: ti,ab,kw (Word variations have been searched)
58. “Inviro SNAP!”: ti,ab,kw (Word variations have been searched)
59. “Maxxon safety syringe*”: ti,ab,kw (Word variations have been searched)
60. “monoject magellan”: ti,ab,kw (Word variations have been searched)
61. “needle-pro”: ti,ab,kw (Word variations have been searched)
62. “point-lok”: ti,ab,kw (Word variations have been searched)
63. “wandplus”: ti,ab,kw (Word variations have been searched)
64. “safetyglide”: ti,ab,kw (Word variations have been searched)
65. “safety wand”: ti,ab,kw (Word variations have been searched)
66. “powder ject”: ti,ab,kw (Word variations have been searched)
67. #1 or #2 or #3 or #4 or #5 or #6 or #7 or #8 or #9 or #10 or #11 or #12
68. #13 or #16 or #17 or #20 or #21 or #22 or #23 or #24 or #25 or #30 or #31 or #32 or #33 or #34 or #35 or #36 or #37 or #38 or #39 or #40 or #41 or #42 or #43 or #44 or #45 or #46 or #47 or #48 or #49 or #50 or #51 or #52 or #53 or #54 or #55 or #56 or #57 or #58 or #59 or #60 or #61 or #62 or #63 or #64 or #65 or #66
69. #67 and #68

Search strategy used in CINAHL:

1. (MH “Health Personnel + ”)
2. (Healthcare or health-care or (health W care)) N2 worker*
3. Paramedic*
4. (medical or nurs*or ancillary) N2 staff*
5. (Medical N2 laboratory N2 techn*)
6. Pharmacist*
7. physician*
8. Hospitalist*
9. internist*
10. doctor*
11. Phlebotomist*
12. (MH “Needlestick Injuries”)
13. (MH “Occupational-Related Injuries”)
14. (MH “Accidents, Occupational”)
15. injur* N3 (syringe* or needle* or inject*)
16. (blood N3 collection) N3 (syringe* or needle* or system* or device* or material* or product* or set*)
17. (need-less or needless or needle-free or needlefree) N3 (syringe* or needle* or system* or device* or material* or product* or set* or inject*)
18. Single N3 “use” N3 (syringe* or needle* or system* or device* or material* or product* or set* or inject*)
19. prevent* N3 (syringe* or needle* or system* or device* or material* or product* or set* or inject*)
20. (MH “Equipment Reuse”)
21. (MH “Disposable Equipment”)
22. (MH “Sharps Disposal”)
23. (MH ”Equipment Safety”)
24. (syringe* or needle* or system* or device* or material* or product* or set* or inject*)
25. S20 OR S21 OR S22 OR S23
26. S24 AND S25
27. Disposable N2 equipment* N3 (syringe* or needle* or inject*)
28. (Disposable N2 equipment*) N3 (syringe* or needle* or inject*)
29. Sharp*
30. (prefill* or pre-fill*) N3 (syringe* or needle* or inject*)
31. Autopen or auto-pen
32. “Vetter Lyo-ject”
33. Vasceze
34. Sterimatic
35. “Safe-Point”
36. “Needle-Pro”
37. Hypak
38. “Slip-lock”
39. Luerlok
40. “Bio-Set”
41. “Auto-disposable syringe*
42. (prefill* or pre-fill*) N2 syringe*
43. “Safety-Lok”
44. “BD Hypak”
45. Kendall’s N2 Monoject
46. “autodestruct syringe”
47. autodestruct w syringe
48. SoloShot
49. “Monodose syringe*”
50. Monodose W syringe*
51. “Unifine pentip*”
52. Autoject
53. ultrasafe N passive N delivery N system
54. “Tip-Lok”
55. “Gettig Guard”
56. “Inviro SNAP!”
57. “Maxxon safety syringe*”
58. “monoject magellan”
59. “needle-pro”
60. “point-lok”
61. “wandplus”
62. “safetyglide”
63. “powder ject”
64. S1 OR S2 OR S3 OR S4 OR S5 OR S6 OR S7 OR S8 OR S9 OR S10 OR S11
65. S12 OR S13 OR S14 OR S15 OR S16 OR S17 OR S18 OR S19 OR S26 OR S27 OR S28 OR S29 OR S30
66. S31 OR S32 OR S33 OR S34 OR S35 OR S36 OR S37 OR S38 OR S39 OR S40 OR S42 OR S43 OR S44 OR S45 OR S46 OR S47 OR S48 OR S50 OR S51 OR S52 OR S53 OR S54 OR S55 OR S58 OR S59 OR S60 OR S61 OR S62 OR S63 OR S65
67. S65 OR S66
68. S64 AND S67.

## Additional files

Additional file 1: Table S1. Table of all screened but excluded studies with their corresponding reasons of exclusion. (DOCX 14 kb)
Additional file 2: Table S2. Table of detailed characteristics of all the included studies (DOCX 41 kb)
Additional file 3: Table S3. Risk of bias assessment table for the included randomized study with its underlying judgments. (DOCX 14 kb)
Additional file 4: Table S4. Risk of bias assessment table for all the other included non-randomized studies with their underlying judgments. (DOCX 27 kb)

## Abbreviations

HBV: Hepatitis B virus
HCV: Hepatitis C virus
HCW: Healthcare workers
HIV: Human immunodeficiency virus
NSI: Needle-stick injuries

## References

[1] Jeffress CN. Occupational Exposure to Bloodborne Pathogens; Needlestick and Other Sharps Injuries; Final Rule. In: Administration OSH, ed. United States Department of Labor Website; 2001. https://www.osha.gov/pls/oshaweb/owadisp.show_document?p_table=FEDERAL_REGISTER&p_id=16265.

[2] Vaughan ARaP. The World Health Report 2002. In: Campanini B, ed. Reducing Risks, Promoting Healthy Life: World Health Organization; 2002.

[3] Panlilio AL, Orelien JG, Srivastava PU (2004). Estimate of the annual number of percutaneous injuries among hospital-based healthcare workers in the United States, 1997-1998. Infect Control Hosp Epidemiol.

[4] McCormick RD, Maki DG (1981). Epidemiology of needle-stick injuries in hospital personnel. Am J Med.

[5] Centers for Disease Control and Prevention (CDC) (1997). Evaluation of safety devices for preventing percutaneous injuries among health-care workers during phlebotomy procedures -- Minneapolis-St. Paul, New York City, and San Francisco, 1993-1995. MMWR Morb Mortal Wkly Rep.

[6] Mendelson M, Solomon R, Shekletski E (1997). Evaluation of safety devices for preventing percutaneous injuries among health-care workers during phlebotomy procedures - Minneapolis-St Paul, New York City, and San Francisco, 1993-1995. J Am Med Assoc.

[7] McCormick RD, Meisch MG, Ircink FG (1991). Epidemiology of hospital sharps injuries: a 14-year prospective study in the pre-AIDS and AIDS eras. Am J Med.

[8] Cardo DM, Culver DH, Ciesielski CA (1997). A case-control study of HIV seroconversion in health care workers after percutaneous exposure. N Engl J Med.

[9] Jagger JPaJ. Reducing sharps injury risk in intensive care settings ADVANCES IN EXPOSURE PREVENTION: International Healthcare Worker Safety Center, University of Virginia; 2005.

[10] Vincent A (2010). 12 self-care steps: your profession is a demanding one. Taking care of yourself is necessary. Massage Ther J.

[11] Alarcon W. Preventing Needlesticks in Surgical Personnel NIOSH Science Blog: Safer Healthier Workers. Centers for Disease Control and Prevention; 2008.

[12] Bloodborne Pathogens Exposure Information Sheet. Occupational Health Services 2013. https://hr.umich.edu/benefits-wellness/health/mhealthy/occupational-health/occupational-health-services/ohs-services/evaluation-exposures-bloodborne-pathogens/bloodborne-pathogens-exposure-information-sheet. Accessed 15 Aug 2014.

[13] Pruss-Ustun A, Rapiti E, Hutin Y (2005). Estimation of the global burden of disease attributable to contaminated sharps injuries among health-care workers. Am J Ind Med.

[14] How to Prevent Needlestick Injuries: Answers to some important questions. In: Labor USDo, ed.: Occupational Safety & Health Administration; 1993. https://www.osha.gov/Publications/osha3161.pdf.

[15] Beekmann SE, Henderson DK (2005). Protection of healthcare workers from bloodborne pathogens. Curr Opin Infect Dis.

[16] Gartner K (1993). Impact of a needleless intravenous system in a university hospital. J Healthc Mater Manage.

[17] Heinrich J, Stark P (2000). Occupational Safety: Selected Cost and Benefit Implications of Needlestick Prevention Devices for Hospitals. United States General Accounting Office.

[18] Garvin M. IV Insertion Safety Devices: Evaluating Both Safety and Clinical Performance. 2001. http://www.infectioncontroltoday.com/articles/2001/07/iv-insertion-safety-devices-evaluating-both-safet.aspx. Accessed December 2014.

[19] Harb AC, Tarabay R, Diab B (2015). Safety engineered injection devices for intramuscular, subcutaneous and intradermal injections in healthcare delivery settings: a systematic review and meta-analysis. BMC Nurs.

[20] Cardo DM, Culver DH, Ciesielski CA (1997). A case-control study of HIV seroconversion in health care workers after percutaneous exposure. Centers for Disease Control and Prevention Needlestick Surveillance Group. N Engl J Med.

[21] Jagger J, Hunt EH, Brand-Elnaggar J (1988). Rates of needle-stick injury caused by various devices in a university hospital. N Engl J Med.

[22] Elie Akl RB, Batoul D, Alain H, Selma K, Rami T (2014). Sharp injury prevention syringes. 10 March 2014 ed. PROSPERO International prospective register of systematic reviews: University of York- Centre for Reviews and Dissemination.

[23] Liberati A, Altman DG, Tetzlaff J (2009). The PRISMA statement for reporting systematic reviews and meta-analyses of studies that evaluate health care interventions: explanation and elaboration. PLoS Med.

[24] Tosini W, Ciotti C, Goyer F (2010). Needlestick injury rates according to different types of safety-engineered devices: results of a French multicenter study. Infect Control Hosp Epidemiol.

[25] Guyatt GH, Oxman AD, Vist G (2011). GRADE guidelines: 4. Rating the quality of evidence--study limitations (risk of bias). J Clin Epidemiol.

[26] Adams D, Elliott TS (2006). Impact of safety needle devices on occupationally acquired needlestick injuries: a four-year prospective study. J Hosp Infect.

[27] Guyatt G OA, Akl EA, Kunz R, Vist G, Brozek J, Norris S, Falck-Ytter Y, Glasziou P, DeBeer H, Jaeschke R, Rind D, Meerpohl J, Dahm P, Schünemann HJ. GRADE guidelines: 1. Introduction-GRADE evidence profiles and summary of findings tables. Elsevier; 2011. 10.1016/j.jclinepi.2010.04.026 21195583

[28] Beason R, Bourguignon J, Fowler D (1992). Evaluation of a needle-free intravenous access system. J Intraven Nurs.

[29] Derevnuk A, Finkelstein-Blond L, Wallach F (2013). Reduction of percutaneous injuries in healthcare workers: Use of a retractable winged steel needle. Am J Infect Control.

[30] Ippolito G, De Carli G, Puro V (1994). Device-specific risk of needlestick injury in Italian health care workers. JAMA.

[31] Kempen PM (1997). Assessing blunt cannulae as replacements for hypodermic needles during intravenous therapy: safety and utility. Infect Control Hosp Epidemiol.

[32] Shimatani M, Matsui Y, Yano K. Comparison of the needlstick injuries due to active and passive design safety intravenous catheters. American Journal of Infection Control Conference: 38th Annual Educational Conference and International Meeting of the Association for Professionals in Infection Control and Epidemiology, Inc, APIC 2011;39(5).

[33] Watters J, MacCallum R, Maurice S (1995). Safelon--a new device to reduce needle-stick injuries during intravenous cannulation. Anaesthesia.

[34] Needlestick-prevention devices. Focus on blood collection devices and catheters. Health Devices. 1998;27(6):184-232. Pubmed PMID: 9669183 9669183

[35] Sossai D, Puro V, Chiappatoli L (2010). Using an intravenous catheter system to prevent needlestick injury. Nurs Stand.

[36] Dubois MC (1991). Protection against the transmission of hematogenous diseases in a hospital milieu. A new device for venous puncture. [French] Protection contre la transmission des maladies hematogenes en milieu hospitalier. Un nouveau materiel de ponction veineuse. Cah Anesthesiol.

[37] Marini MA, Giangregorio M, Kraskinski JC (2004). Complying with the Occupational Safety and Health Administration’s Bloodborne Pathogens Standard: Implementing Needleless Systems and Intravenous Safety Devices. Pediatr Emerg Care.

[38] Waclawski ER (2004). Evaluation of potential reduction in blood and body fluid exposures by use of alternative instruments. Occup Med.

[39] News & views. Safety devices reduce phlebotomy injuries… report by the Centers for Disease Control and Prevention (CDC) (MMWR. 1997;46:21-25). Laboratory Medicine 1997;28(5):293-93.

[40] Safety device evaluations produce efficacy data. Hospital Employee Health. 1997;16(4):40-43.

[41] Bohony J (1993). Fighting the needlestick battle without needles… needleless intravenous piggyback (IVPB) systems. Medsurg Nurs.

[42] Keuren MV, Cunningham C, Hackman B (1992). Impact of a needleless system (NLS) for connecting intravenous tubing on the incidence of needlestick injuries. Am J Infect Control.

[43] Buerke B, Puesken M, Mellmann A (2011). Automatic MDCT injectors: Hygiene and efficiency of disposable, prefilled, and multidosing roller pump systems in clinical routine. Am J Roentgenol.

[44] Casey AL, Elliott TS (2007). The usability and acceptability of a needleless connector system. Br J Nurs.

[45] Ihrig M, Cookson ST, Campbell K (1997). Evaluation of the acceptability of a needleless vascular-access system by nurses. Am J Infect Control.

[46] Sibbitt RR, Palmer DJ, Sibbitt WL (2008). Integration of patient safety technologies into sclerotherapy for varicose veins. Vasc Endovasc Surg.

[47] Suzuki R, Kimura S, Shintani Y (2006). The efficacy of safety winged steel needles on needlestick injuries. Kansenshogaku zasshi.

[48] Catalan Gomez MT, Sol Vidiella J, Castella Castella M (2010). Implementation of safety devices: biological accident prevention. [Spanish] Implantacion de material de biosegurida: prevencion de accidentes biologicos. Revista de enfermeria (Barcelona, Spain).

[49] Roudot-Thoraval F, Montagne O, Schaeffer A (1999). Costs and benefits of measures to prevent needlestick injuries in a university hospital. Infect Control Hosp Epidemiol.

[50] Griswold S, Bonaroti A, Rieder CJ, et al. Investigation of a safety-engineered device to prevent needlestick injury: Why has not StatLock stuck? BMJ Open 2013;3(4) doi:10.1136/bmjopen-2012-002327. 10.1136/bmjopen-2012-002327 PMC3641494 23616435

[51] Reddy SG, Emery RJ (2001). Assessing the effect of long-term availability of engineering controls on needlestick injuries among health care workers: a 3-year preimplementation and postimplementation comparison. Am J Infect Control.

[52] Theou-Anton N, Sinegre M, Gasciolli S (2009). Quality and safety improvements in the preparation and administration of intravenous cytotoxic therapies with new medical devices. EJHP Pract.

[53] Edwards C, Johnson C (2012). Evaluation of a luer-activated intravenous administration system. JAVA.

[54] Gartner K (1992). Impact of a needleless intravenous system in a university hospital. Am J Infect Control.

[55] Gershon RRM, Pearse L, Grimes M (1999). The impact of multifocused interventions on sharps injury rates at an acute-care hospital. Infect Control Hosp Epidemiol.

[56] L’Ecuyer PB, Schwab EO, Iademarco E (1996). Randomized prospective study of the impact of three needleless intravenous systems on needlestick injury rates. Infect Control Hosp Epidemiol.

[57] Lawrence LW, Delclos GL, Felknor SA (1997). The effectiveness of a needleless intravenous connection system: an assessment by injury rate and user satisfaction. Infect Control Hosp Epidemiol.

[58] MacPherson J (1996). The interlink needleless intravenous system did not reduce the number of needlestick injuries in Christchurch hospital operating theatres. N Z Med J.

[59] Orenstein R, Reynolds L, Karabaic M (1995). Do protective devices prevent needlestick injuries among health care workers?. Am J Infect Control.

[60] Skolnick R, LaRocca J, Barba D (1993). Evaluation and implementation of a needleless intravenous system: making needlesticks a needless problem. Am J Infect Control.

[61] Terrell F, Williams B (1993). Implementation of a customized needleless intravenous delivery system. J Intraven Nurs.

[62] Wolfrum J (1994). A follow-up evaluation to a needle-free I.V. system. Nurs Manag.

[63] Yassi A, McGill ML, Khokhar JB (1995). Efficacy and cost-effectiveness of a needleless intravenous access system. Am J Infect Control.

[64] Mendelson MH, Short LJ, Schechter CB (1998). Study of a needleless intermittent intravenous-access system for peripheral infusions: analysis of staff, patient, and institutional outcomes. Infect Control Hosp Epidemiol.

[65] Billiet LS, Parker CR, Tanley PC, Wallas CH (1991). Needlestick injury rate reduction during phlebotomy: a comparative study of two safety devices. Lab Med.

[66] McCleary J, Caldero K, Adams T (2002). Guarded fistula needle reduces needlestick injuries in hemodialysis. Nephrol News Issues.

[67] Rogues A, Verdun-Esquer C, Buisson-Valles I (2004). Impact of safety devices for preventing percutaneous injuries related to phlebotomy procedures in health care workers. Am J Infect Control.

[68] Hoffmann C, Buchholz L, Schnitzler P (2013). Reduction of needlestick injuries in healthcare personnel at a university hospital using safety devices. J Occup Med Toxicol.

[69] Lamontagne F, Abiteboul D, Lolom I (2007). Role of safety-engineered devices in preventing needlestick injuries in 32 French hospitals. Infect Control Hosp Epidemiol.

[70] Sohn S, Eagan J, Sepkowitz KA (2004). Effect of implementing safety-engineered devices on percutaneous injury epidemiology. Infect Control Hosp Epidemiol.

[71] Valls V, Lozano MS, Yanez R (2007). Use of safety devices and the prevention of percutaneous injuries among healthcare workers. Infect Control Hosp Epidemiol.

[72] Whitby M, McLaws M, Slater K (2008). Needlestick injuries in a major teaching hospital: the worthwhile effect of hospital-wide replacement of conventional hollow-bore needles. Am J Infect Control.

[73] Elder A, Paterson C (2006). Sharps injuries in UK health care: a review of injury rates, viral transmission and potential efficacy of safety devices. Occup Med.

[74] Lavoie MC, Verbeek JH, Pahwa M (2014). Devices for preventing percutaneous exposure injuries caused by needles in healthcare personnel. Cochrane Database Syst Rev.

[75] Mustafa RA, Santesso N, Brozek J (2013). The GRADE approach is reproducible in assessing the quality of evidence of quantitative evidence syntheses. J Clin Epidemiol.

[76] Guyatt GH, Oxman AD, Sultan S (2011). GRADE guidelines: 9. Rating up the quality of evidence. J Clin Epidemiol.

