# Supplementary material for: Use of safety-engineered devices by healthcare workers for intravenous and/or phlebotomy procedures in healthcare settings: a systematic review and meta-analysis
Source: BMC Health Serv Res. 2016 Sep 1;16:458. doi: 10.1186/s12913-016-1705-y (PMC5007867; doi:10.1186/s12913-016-1705-y)
Supplement: Additional file 1: Table S1. — Table of all screened but excluded studies with their corresponding reasons of exclusion. (DOCX 14 kb) [file 12913_2016_1705_MOESM1_ESM.docx]

| **Study Name (Author, Year)** | **Reason of Exclusion** |
| --- | --- |
| Lulloff 1996 | Lacks a control or standard of reference |
| Griswold 2013 | Reports economic analysis and cost-relevant data exclusively (no data about NSI) |
| Dubois 1991 | Lacks an actual intervention (Commentary) |
| Gomez 2010 | Lacks data for pre- or post- intervention period (pre) |
| Buerke 2011 | Reports data on device practicality (speed of preparation and injection) |
| Theou-Anton 2009 | Implemented device is a non-safety device |
| Kempen 1997 | Lacks sufficient data of study design and population |
| Marini 2004 | Lacks an actual intervention (Commentary) |
| Shimatani 2011 | Lacks a control or standard of reference |
| Bohony 1993 | Non-original research paper (Review) |
| Casey 2007 | Reports data on HCW evaluation of devices |
| Beason 1992 | Lacks a control or standard of reference |
| Ihrig 1997 | Reports data on HCW evaluation of devices (nurses’ opinion) |
| Watters 1995 | Lacks a control or standard of reference |
| Sibbitt 2008 | Reports data on HCW evaluation of devices |
| Health Devices 1998 | Lacks an actual intervention (Commentary) |
| Ippolito 1994 | Lacks a control or standard of reference |
| Waclawski 2004 | Lacks an actual intervention (Observation) |
| Puro 2010 | Lacks an actual intervention (Commentary) |
| Reddy 2001 | Not reporting data separately for different procedures |
| Derevnuk 2013 | Lacks a control or standard of reference |
| Hospital Employee Health 1997 | Non-original research paper (Magazine article) |
| Laboratory Medicine 1997 | Non-original research paper (Magazine article) |
| Suzuki 2006 | Lacks data for pre- or post- intervention period (post) |
| Roudot-Thoraval 1999 | Lacks sufficient data of study design and population |
| Van Keuren 1992 | Non-original research paper (Abstract) |
