# Supplementary material for: Use of safety-engineered devices by healthcare workers for intravenous and/or phlebotomy procedures in healthcare settings: a systematic review and meta-analysis
Source: BMC Health Serv Res. 2016 Sep 1;16:458. doi: 10.1186/s12913-016-1705-y (PMC5007867; doi:10.1186/s12913-016-1705-y)
Supplement: Additional file 2: Table S2. — Table of detailed characteristics of all the included studies (DOCX 41 kb) [file 12913_2016_1705_MOESM2_ESM.docx]

| Study Name | Device | Study Design | Participants/ Setting | Intervention | Control | Outcomes |
| --- | --- | --- | --- | --- | --- | --- |
| **Gartner 1992**  **Funding: *not reported***  **Conflict of interest: *not reported***  **Data Collection: *Retrospective for “Pre”***  ***vs***  ***Prospective for “Post”*** | - Needleless IV system (Interlink IV System, Baxter Healthcare Corp., Parenterals Div., Deerfield, Ill.) with 2 basic   components: a blunt plastic cannula, which replaces the conventional steel needle, and an injection port.  IV system is accessed by inserting the cannula into the injection port. A needle is still necessary to pierce the patient's skin for initial insertion | - A retrospective (partly prospective) study with data collected by 6-month intervals - Pre: previous 5 years: January 1985 to August 1990. (retrospectively collected) - Post: 6 months after the implementation of the system: March to August 1990 (prospectively collected) | - Nursing staff members at Montefiore University Hospital (500-bed acute care facility), part of the University of Pittsburgh Medical Center | - Implementation of Needleless IV system in Jan 1990. - Nursing staff   members were educated during a 2-month period, from January to February 1990 | “conventional steel needles” | Incidence of NSI (self-reported; a report generated each month listed all employee exposures) |
| **Skolnick 1993**  **Funding: *not reported***  **Conflict of interest: *not reported***  **Data Collection: *Unclear*** | - Baxter InterLink IV Access System (Baxter Healthcare Corp., Deerfield,Ill.),   consisting of blunt plastic cannulas that insert into specially designed injection sites. | - Pre-post study - Pre: 8 months January 1990-August 1990; post: 8 months January 1991-August 1991). | - No specific information provided   Olive View Medical Center, UCLA, US | - Implementation of a new IV systems in January 1991 - The educational campaign 3 weeks before implementation (posters, videotape   demonstrating use of the system presented to  all nurses and a resource nurse) | “Traditional IV system” which uses needles to deliver medications. | Incidence of NSI among HCWs (unspecified means of reporting) |
| **Edwards 2012**  **Funding: *An educational grant from Baxter***  **Conflict of interest: *not reported***  **Data Collection: *Retrospective Throughout*** | - Luer-activated IV administration   system | - Retrospective,   Descriptive evaluation study.   - Pre and post intervention analysis of NSI rates (pre: January 2005 to May 2010, post: June 2010 to September 2011) | 874 frontline nursing staff members at Thunder Bay Regional Health Sciences Centre, Ontario, Canada | - Implementation of Luer-activated IV administration   system | Split-septum IV administration sets | Incidence of NSI (self-reporting to Occupational Health and Safety Department of the center along with surveys collected from the nurses) |
| **Yassi 1995**  **Funding: *not reported***  **Conflict of interest: *not reported***  **Data Collection: *Unclear*** | A needleless intravenous access system (Interlink IV access system, Baxter Healthcare Corp., Parenterals IV system Division, Deerfiled III and Beckton-Dickinson Canada, Inc. Mississauga, Ontario, Canada) | - Most likely retrospective design - Pre: October 1991 through September 1992. - Post: October 1992 through September 1993. | Health Science Center (HSC), a 1100-bed tertiary care hospital in the inner core of Winnipeg, Manitoba, Canada  Employs approximately 6000 workers | - Introduction of the Interlink products - The company representative gave in-service instruction to nurses on all wards about the use of Interlink products before commencement of the study | Conventional heparin-lock intermittent intravenous system | Incidence of NSI (prompt self-reporting to the department of Occupational and Environmental medicine that record the injury in injury reports) among HCWs |
| **MacPherson 1996**  **Funding: *not reported***  **Conflict of interest: *not reported***  **Data Collection: *Unclear*** | Baxter Interlink Needleless IV system | - Pre-post study (pre: December 1992-May 1993, post: July-December 1994) | Nurses, doctors and hospital aids at Christchurch Hospital Operating theater(530-bed teaching hospital); no further details provided | Needleless IV system was implemented in July 1994 | Unspecified devices | Incidence of NSI (data obtained from the infection control officer at Canterbury Health Laboratory) |
| **L’Ecuyer 1996**  **Funding: *not reported***  **Conflict of interest: *not reported***  **Data Collection: *Prospective Throughout*** | - 3 tubing needleless IV systems   -Metal blunt cannula (MBC Lifeshield Abbott Laboratories Chicago, IL).  -Plastic blunt cannula (PBC; Interlink, Baxter/BD, Deerfield, IL).  -Plastic two-way valve system (2-way; Saf-site Burron Medical Bethlehem, PA). | - Randomized controlled trial (with two experimental and two controlled groups) - From June 1992 through March 1994 | - 223 nurses from general medical, general surgical and intensive care units (15% of all nursing divisions). - 1000 bed tertiary care Midwestern hospital, Barnes Hospital affiliated with Washington University School of Medicine in St Louis, Missouri | - Introduction of 3 tubing needleless IV systems in 3 study areas (MBC was used for only 4 months then replaced by PBC) - Participating nursing divisions received needle safety education. - “Device-training was provided to the experimental groups only” | - “Traditional needled device (Standard needle system)” in 3 controlled areas | •Incidence of NSIs (Self reporting; an infection control practitioner visited participating divisions to check NSI logs) |
| **Lawrence 1997**  **Funding: *Funded in part by the Educational Resource Centers, Inc, and the National Institute for Occupational Safety and Health***  **Conflict of interest: *not reported***  **Data Collection: *Retrospective Throughout*** | - Intravenous Connection System (InterLink Needleless) consisting of   blunt plastic cannulas and compressed latex injection sites to make connections in the IV system (Baxter  Healthcare Corp, Round Lake, IL) | - Retrospective data collection - Pre-post intervention design (pre: 1989 to 1991, post: throughout 1993) | - 7500 staff in two tertiary-care teaching hospitals, one general (4,300) and one pediatric hospitals (3200), located in a large metropolitan medical center in Houston, Texas. | - Implementation of an needeless IV connection system in 1992 - Education provided to all personnel using the device   Compliance with use of the device was monitored informally by each individual unit manager | Unspecified needle devices | Incidence of NSI among HCWs (self reported injuries entered into a computerized database; injury reports forwarded to employee health department and a phone call was placed within 24 hours) |
| **Mendelson 1998**  **Funding: *Supported by the grant “Pilot Study of Needlestick Prevention Devices”, New York State Department of Health and Braun Medical, Inc.***  **Conflict of interest: *not reported***  **Data Collection: *Retrospective for “Pre”***  ***vs***  ***Prospective for “Post”*** | A needleless intermittent access system (Safesite; Braun Medical Inc Bethlehem, PA).  Consists of one-way reflux valve connected to extension tubing that fits directly into the hub of an intravenous catheter. The rubber port is incompatible with the needle. | - Cross-over non-randomized trial - 6 months period (July - December 1991) - Phase I: 13 weeks; cross over; phase II: 12 weeks | “All staff who utilized, manipulated, or may have been exposed to sharps on study and control units”   - 16 medical and surgical units of the Mount Sinai Medical center at a 1100 bed acute care teaching referral medical center in New York City. - “Pediatrics, obstetrics-gynecology, and intensive-care units were excluded” | - Implementation of needleless intermittent intravenous access system.   “At the start of each study phase, education was provided to nursing personnel on the study and control unit… In addition, study-unit personnel received direct hands-on training with NL” | Conventional Heparin-lock system (CHL)  “At the start of each study phase, education was provided to nursing personnel on the study and control unit” | - Incidence of NSI (self-reported and registered into both standardized forms and the hospital’s injury log) among HCWs |
| **Terrell 1993**  **Funding: *not reported***  **Conflict of interest: *not reported***  **Data Collection: *Unclear*** | - IV tubing that does not have latex injection ports but instead an open port with a luer lock cap with a reflux valve added to the open ports to prevent fluid leakage and having to clamp the tubing before use | - Pre and Post design - Pre: throughout 1992 - Post: Feb-April 1993 | - Staff nurses and IV nurses in all departments at two hospitals: Kennestone Hospital and Windy Hill Hospital, in Marietta, GA. | - Implementation of a customized needleless IV delivery system on Feb 1993 - Classes (30min for 16 hours/day for a week) given to all hospital departments on the use of the new custom device in Jan 1993 | - Formerly existent IV sets with latex injection ports. | - Incidence of NSI among HCWs (immediate self-reporting of NSI). |
| **Orenstein 1995**  **Funding: *Partial support by grant from Baxter Healthcare Corp.***  **Conflict of interest: *not reported***  **Data Collection: *Prospective Throughout*** | The Baxter  InterLink intravenous system (Baxter Healthcare  Corp., Deerfield, Ill.) components (connector  loop with male Luer slip adapter, threaded lock  cannula, lever lock cannula, InterLink cannula,  InterLink injection site, and Y-lock cannula) | - Prospective controlled study with a pre and post intervention analysis of NSI rates with a standardized questionnaire from January 14, 1992 through January 14, 1993 (the “pre” period is the first 6 months, “post” period is the following 6 months) | - 262 nursing personnel, including the registered nurses, licensed practical nurses, nursing aides, and students, as well as medical teams consisting of an attending physician, resident physician, interns, and medical students on the study units   “ Six hospital inpatient units, consisting of three medical units, two surgical units, and a 15-bed surgical-trauma intensive care unit” at the 900-bed urban tertiary care referral center for central Virginia- Medical College of Virginia Hospital, USA | - Implementation of a needleless IV system to four randomly chosen study units and the surgical-trauma intensive care unit (units A through D and ICU) in July 1992   +  “An extensive in-service educational program was  conducted by the manufacturer and reinforced by  the unit nursing managers” | - “Traditional” Devices   (Unspecified) | Incidence of NSI (“Data on occupational needlestick exposures  were actively captured by three mechanisms: (1)  infection control practitioners visited  the study units weekly and collected standardized  personnel exposure forms from the nursing  supervisors on these units; these forms were completed  by each HCW at the time of an occupational  NSI; a physician investigator collected  forms weekly from the house staff teams on the  study units for each NSI; (2) Employee health  records were reviewed daily to capture any NSIs  reported to the employee health service as mandated  but not to the study personnel. (3) A hospital  computer system pathway used to order laboratory  serologic tests for HIV-1 and hepatitis B infections  after occupational exposure was reviewed  weekly, and HCWs with NSIs on the study units  who failed to report the injury on the study questionnaire  were contacted to complete the questionnaire”) among HCWs |
| **Hoffman 2013**  **Funding: *not reported***  **Conflict of interest: *“The authors declare that they have no conflicts of interest”***  **Data Collection: *Unclear*** | - The safety mechanism was triggered actively for the hypodermic needles while passively for the lancets | - Most likely retrospective study - Pre and post intervention analysis of NSI rate (1 year before and 2 year after; 2007-2009). | - 6493 and 6683 full-time healthcare personnel in 2007 and 2009 respectively. Included nurses, doctors, lab technicians and students) at the University Hospital Heidelberg in southwestern Germany | - Implementation of the safety devices throughout the hospital with simultaneous mandatory training for all healthcare personnel in 2008 | - Unspecified needle devices | - Incidence of NSI (self-reported) amongst HCW |
| **Valls 2007**  **Funding: *Funds from “Direccion General***  ***de Salud Publica de la Generalitat Valenciana”—“Comunidad Autonoma***  ***de Valencia”—Spain***  **Conflict of interest:**  ***“All authors report no conflicts of interest relevant to this article”***  **Data Collection: *Prospective Throughout*** | - Safety-engineered devices included the following:   vacuum phlebotomy systems, blood-gas syringes, lancets (with retractable, single-use puncture sticks), and intravascular catheters | - Prospective “quasi experimental” trial - Before-and-after intervention evaluation. Pre: October 2004 - March 2005; Post: October 2005 - March 2006. | - 75 nurses working on selected (and not all) wards of Hospital Virgen de la Salud-Elda; Alicante, Spain | - Safety-engineered devices introduced in October 2005 - Nurses participated in a 2-hour training experience about the devices to be tested and ways to improve compliance in both the use of the devices and the reporting of injuries - An additional 15 minutes of on-site training was carried out each time a new device was tested. | - Unspecified “conventional devices” | - Incidence of NSI (voluntary self-reporting complimented with active surveillance and reporting by nurses in charge) among HCWs |
| **Whitby 2008**  **Funding: *not reported***  **Conflict of interest: *not reported***  **Data Collection: *Prospective Throughout*** | - Safety engineered devices, including needle-free intravenous systems, and safety winged butterfly needles | - Prospective study design - Before and after collection of data on NSI; Pre: 2000-2004; post: 2005-2006 | - Full-time employee and part-time HCW from multiple occupational groups within the hospital including medical, nursing, hotel services, and other in an 800-bed university teaching - Princess Alexandra Hospital, Brisbane, Australia | - Introduction of the safety engineered devices | - Unspecified needle devices | - Risk of needle stick injury - Risk of blood stream infections |
| **Sohn 2004**  **“Effect of implementing safety-engineered devices”**  **Funding: *National Institute of Allergy and Infectious Diseases at the NIH; the Centers for Disease Control and Prevention; the National surveillance system for hospital healthcare workers at the CDC; and the Prevention Epicenters Program***  **Conflict of interest: *not reported***  **Data Collection: *Prospective Throughout*** | - “Safer-needle system” was composed of “variety of safety-engineered devices from seven manufacturers to allow for needle-safe intravenous (IV) delivery, blood collection, IV insertion, and intramuscular and subcutaneous injection” | - Prospective study - Before-and-after intervention trial: before: Jan 1998–Dec 2000; after: Feb 2001– Jan 2002 | - Staff (about 4000 FTEs per year) at a 427-bed, tertiary-care hospital in Manhattan | •Implementation of the “safer-needle system”  •All HCWs responsible for direct patient care were required to attend a training session in the months preceding implementation | - Unspecified “conventional devices” | Incidence of percutaneous injuries (self-reported) |
| **Wolfrum 1994**  ***A follow-up evaluation to a needle-free IV system***  **Funding: *not reported***  **Conflict of interest: *not reported***  **Data Collection: *Retrospective Throughout*** | Needle free IV system:  All plastic material.  “its 2 way valve replaces rubber injection caps for IV devices and opens by inserting any standard syringe for IV tubing.” | Retrospective records from 1989-1992. | Employees( physicians, nurses, technician, laboratory, housekeeping) within a 394-bed university hospital in Wisconsin (some inpatient and outpatient clinics). | Implementation of needle free system in 1991.  Implementation of the needle free system took 3 weeks in 1991.    “Employee who received training following guidelines of the occupational safety and health administration (OHSA).” | “Conventional phlebotomy nonsafety devices” | NSIs (Self-reported exposures). |
| **Gershon 1999**  **Funding:**  ***Financed by the Centers for Disease Control and Prevention/National Institute of Occupational Safety and***  ***Health; Mr. William E. Flanagan, Jr.***  **Conflict of interest: *not reported***  **Data Collection: *Prospective Throughout*** | Needleless IV therapy system | Pre and post intervention study:  “Pre”: 2-year period 1990 through 1991.  “Post”: 6-years period 1993 through 1998. | “HCWs (clinical, laboratory, ancillary workers, nurses, technicians, support staff)”  “Physicians and house staff were not included because of difficulty determining a consistent denominator”  2300 employees in a 450 bed mid-sized acute-care community hospital in Greater Washington DC. | Implementation of a needless IV system in 1992  “Accompanied by facility-wide training programs, unit-specific training, and train-the-trainer programs aimed at managers.”  “Administrative controls also were implemented: an intensive educational effort including  mandatory annual training in general infection  control and sharps injury prevention issues for all hospital  employees; employee health programs were  enhanced and expanded; and the injury reporting  process was expedited, and employee concerns regarding  confidentiality were identified and addressed” | “Alternative systems” | Incidence of NSIs among HCWs (self-reported via a detailed exposure questionnaire) |
| **La Montagne 2007**  **Funding: *Financially supported by the French Ministry of Health. Additional***  ***financial support was provided by la Mutuelle Nationale des***  ***Hospitaliers and the following companies: Becton-Dickinson, Bristol-Myers-***  ***Squibb, Glaxo Wellcome, Johnson & Johnson Medical, Kendall Sherwood***  ***David & Geck, MAPA Hutchinson, Merck Sharp & Dohme Chibret, Sanofi***  ***Winthrop, SIMS France, and Terumo***  **Conflict of interest: *not reported***  **Data Collection: *Prospective Throughout*** | SEDs were defined  as devices that include an integrated safety feature, ensure  immediate and automatic deployment of the safety feature,  and allow single-handed, irreversible activation of the safety  feature by the user.  Three types of devices :  catheters for intravenous infusion, winged steel needles used  for infusion or blood collection, and vacuum-tube blood-collection devices. | Multicenter prospective survey with a 1-year follow-up period (from April 1999 through March 2000). | A total of 1,506 nurses in medical or intensive care units in a total of 102 medical units and intensive care units from 32 hospitals in France which represented approximately 2,000 beds.  These hospitals included 29 public institutions (11  tertiary care hospitals and 18 secondary care hospitals) and  3 private institutions in various regions in France | Implementation of SEDs in 1999 | Conventional devices | Incidence of NSIs (self-reported into a notebook at each unit and blinded  for analysis) among HCWs |
| **McCleary 2002**  **Funding: *not reported***  **Conflict of interest: *not reported***  **Data Collection: *Retrospective for “Pre”***  ***vs***  ***Prospective for “Post”*** | - Arteriovenous Fistula needle (MasterGuard Anti-Stick needle protector, Medisystems corporation)   This guarded AVFN consists of a slotted guard attached to a conventional winged medisystems AVFN. The Guard is separate from the winged AVFN and thus permits standard cannulation and site selection. The Guard is activated by the pressure-holding fingers. | - A retrospective (partly prospective) study - Pre and post study design with pre: retrospective collection of NSI rates 15 months starting Jan 1, 1999; post: prospective collection of NSI rates for 9 months starting April 1, 2000 | 107 clinical staff and 273-331 patients involved in retrospective period  128 clinical staff personnel and 331-370 patients involved in prospective period.  At 5 affiliated Dialysis clinics in the Pacific Northwest. | - Implementation of a Guarded AVFN - HCWs trained in the facility’s NSI reporting policy and standard precautions policy - Training in the proper use of the guarded AVFN in the prospective period (10-minute in-vivo training video provided by the manufacturer) | Conventional large-gauge, hollow-bore Unguarded Arteriovenous Fistula needle | Incidence of NSI (No information on how NSI was reported) |
| **Mendelson** **1997**  **Funding: *not reported***  **Conflict of interest: *not reported***  **Data Collection: *Unclear*** | - Each hospital selected evaluate a safe needle device they select: - -Bluntable vacuum-tube blood collection needle activated in the patients’ vein (Puncture-Guard from Bio-Plexus in Tholland, CT) used in 3 hospitals. - - A vacuum-tube blood collection needle with a hinged recapping sheath (Venipuncture Needle-Pro from Smith Industries [Concord Portex] in Keen, NH) used in 4 hospitals. - -Resheathable winged steel needle (Safety-Lok from Becton Dickinson in Franklin Lkaer, NJ), used in 6 hospitals. - Each product requires HCWs to activate the safety devise during or after phlebotomy. | - Two phases study at six university-affiliated urban hospital (3 in Minneaplis-St.Paul, 1 in New York City, and 2 in San Francisco), from 1993-1995. - Phase I (pre): 10 months ranges between 9-12 months where the conventional device was used. - Phase II (post): 12 months ranges between 6-15 months where the conventional device were replaced with the safety devices | - 1699 HCW responds to the survey in phase I and 1421 in phase II. Response rate (75%) - Workers received comprehensive training program that includes “hands-on” experience with the equipment. | - Safety device was implemented in February 1994. | - “Conventional phlebotomy devices” | - Incidence of NSI (data was collected via HCW survey) |
| **Adams 2006**  **Funding: *Educational***  ***grant by Becton Dickinson, Oxford, UK***  **Conflict of interest: *not reported***  **Data Collection: *Prospective Throughout***  Data not provided separately for different devices | - Safety needle devices consisted of hypodermic needle devices with safety feature requiring activation: Safety Glide needles, SafetyGlide TNT insulin units, blunt fill cannulae (Becton Dickinson, Temse, Belgium) - The needles and the blunt fill cannulae appear to be for phlebotomy use | - Prospective cohort study - A four-year (2001-2004), pre and post intervention analysis of NSI rate | All HCW from 4 clinical areas (2 surgical, 1 medical and 1 outpatient) at  the University Hospital Birmingham NHS Foundation Trust (number of participants not reported) | - 2002: enhanced training program - 2003:pilot evaluation of the safety needle devices   Late 2003 throughout 2004: Introduction of the safety needle devices | “Standard hypodermic needles” (2001); although standard needles used in2002 and 2003 there were co-interentions during that year (enhanced training and pilot evaluation) | - Incidence of NSI (data collected prospectively 2001 onwards to the   Trust’s occupational health and safety department and risk management. |
| **Rogues 2004**  ***Impact of safety devices for preventing***  ***percutaneous injuries related to***  ***phlebotomy procedures in health***  ***care workers***  **Funding: *not reported***  **Conflict of interest: *not reported***  **Data Collection: *Prospective Throughout*** | “re-sheathable winged steel needles (SafetyLok, Becton  Dickinson) and Vacutainer blood-collecting tubes with recapping sheaths (SafetyLok, Becton Dickinson).”  “Each  product required the HCW to activate the safety feature  immediately after phlebotomy. The 2 safety mechanisms  required a 2-handed activation.”  “These  devices were used for blood drawing, not for peripheral  intravenous infusion.” | Before-and-after intervention trial over a 7 year period 1993-1999***.***  “Pre”: 3-years period 1993 throughout 1995.  “Post”: 3-years period 1997 throughout 1999. | HCWs  in a 3600-bed university hospital in France. | Implementation of two safety devices in June 1996.  “The safety device company instructed nurses, with sessions and a pamphlet in each ward, on how to activate the safety mechanism following removal of the needle from the patient” | “Conventional phlebotomy non-safety devices” | Incidence of NSIs among HCWs  (self-reported) |
| **Billiet 1991**  **Funding: *not reported***  **Conflict of interest: *not reported***  **Data Collection: *Retrospective for “Pre”***  ***vs***  ***Unclear for “Post”*** | “Safety needle recapping device” and “Shielded blood needle adapter ,Saf-T Clik” | - Prospective study design - Before and after: pre intervention (9 months) June 1987 - March 1988; post intervention (17 months) April 1988-Septmenber 1989 (recapping device: April 1988-December 1988; Saf-T Clik): Jan 1989- September 1989) | 39 phlebotomists in the phlebotomy laboratory of the Vanderbilt University Medical Center | Implementation of two phlebotomy devices | “Standard reusable phlebotomy device with disposable needles” | NSIs in phlebotomy  (Based on review of the “employee incidence records from VUMC pathology department). |
