# Supplementary material for: Use of safety-engineered devices by healthcare workers for intravenous and/or phlebotomy procedures in healthcare settings: a systematic review and meta-analysis
Source: BMC Health Serv Res. 2016 Sep 1;16:458. doi: 10.1186/s12913-016-1705-y (PMC5007867; doi:10.1186/s12913-016-1705-y)
Supplement: Additional file 3: Table S3. — Risk of bias assessment table for the included randomized study with its underlying judgments. (DOCX 14 kb) [file 12913_2016_1705_MOESM3_ESM.docx]

| **Study**  **Name** | **Random sequence generation** | **Allocation concealment** | **Blinding** | **Completeness of data** | **Selective outcome reporting** | **Other** |
| --- | --- | --- | --- | --- | --- | --- |
| **L’Ecuyer 1996**  **Funding: *not reported***  **Conflict of interest: *not reported***  **Data Collection: *Prospective throughout*** | -Unclear risk  Not reported | - Unclear risk  Not reported | - Unclear risk  Not reported | -Unclear risk  Authors did not report any missing data. | - Unclear risk | -High risk  Cross over: “We detected needlestick injuries in study areas during intravenous-therapy-related activities due to continued use of traditional needled devices”  - No mention of Intention to treat analysis |
