# Supplementary material for: Use of safety-engineered devices by healthcare workers for intravenous and/or phlebotomy procedures in healthcare settings: a systematic review and meta-analysis
Source: BMC Health Serv Res. 2016 Sep 1;16:458. doi: 10.1186/s12913-016-1705-y (PMC5007867; doi:10.1186/s12913-016-1705-y)
Supplement: Additional file 4: Table S4. — Risk of bias assessment table for all the other included non-randomized studies with their underlying judgments. (DOCX 27 kb) [file 12913_2016_1705_MOESM4_ESM.docx]

| Study Name | Developing and applying appropriate eligibility criteria | Measurement of intervention | Measurement of outcome | Controlling for confounding | Completeness of data |
| --- | --- | --- | --- | --- | --- |
| **Gartner 1992** | -Low risk  Participants of the Pre-Post periods belong to the same population | -Low risk  The needleless system was introduced throughout the institution.  And during the training period “transitional period” the injuries incurred during this period were not used in determining the impact of the new system. | - Low Risk  “The reporting system remained constant throughout the study, where a report is generated each month listing all employee exposures caused by sharp devices” | - Unclear risk  No information on how confounding was handled. | - Unclear Risk.  Authors did not report any missing data |
| **Skolnick 1993** | -Low risk  Participants of the Pre-Post periods belong to the same population | -Low risk  Old products were removed from all departments and the new products were in place on implementation day. | -Unclear risk  No data on how the NSIs were reported. | - Unclear risk  No information on how confounding was handled. | - Unclear Risk.  Authors did not report any missing data |
| **Edward 2012** | - High risk  Participants of the pre-post periods belong to the same population but response rate was low.  -High risk  “15 months of post-conversion needlestick data vs. 66 months  of pre-conversion needlestick data” | -Unclear risk  No data shows that all conventional IV systems were removed in the study period. | -Unclear risk  No data was given on how the NSI was reported. | -Unclear risk  No information on how confounding was handled. | -Low risk  Authors did not report any missing data |
| **Yassi 1995** | -Low risk.  Participants selected from the same population in the pre and post period | -Unclear risk  No data shows that all conventional IV systems were removed in the study period.  “Some wards continued to use the old ports with pre-Interlink injection sites, which were not intended to be punctured with the blunt cannula” | -Unclear risk  “All NSI are to be reported promptly to the department of occupational and environmental medicine. HCW who incurred an exposure while using an interlink product was personally interviewed by a department of occupational and environment medicine”. | - Unclear Risk  No information on how confounding was handled. | - Unclear Risk  Authors did not report any missing data. |
| **Macpherson 1996** | -Low risk  Participants of the Pre-Post periods belong to the same population | -Unclear risk  No data shows that all conventional IV systems were removed in the study period. | -High risk  “Many potential exposures do however go unreported” | - Unclear risk  No information on how confounding was handled. | - Unclear Risk.  Authors did not report any missing data |
| **Lawrence 1997** | -Low risk  Participants of the pre-post periods belong to the same population | -Low risk  “Compliance with use of the device was monitored informally by each individual unit manager”; data from 1992 (first year of implementation) were not included “to assure distribution to all hospital units and to allow employees to become familiar with the use of the new system” | -Low risk  Three separate data sources were compiled and compared | -Unclear risk  “There are numerous other factors that may have influenced the injury rate in the postintervention year, such as increased education and awareness of risk among HCWs, increased reporting of injuries, and the passage of the OSHA Bloodborne Pathogens Standard in 1991” | -Low risk  While missing data is reported for other variables, none is reported for NSI rates |
| **Mendelson 1998** | -Low risk.  Participants were involved in both arms (cross-over design) | -High risk  Concern about contamination between study arms due to cross over design; e.g., no data showing that all safety devices introduced in the first phase were removed from the corresponding arm in the second phase | -Low risk  A standardized form was used to collect details concerning reported percutaneous injuries and associated devices and procedures. The hospital (OSHA) injury log was used to verify that all reported percutaneous injuries were included. | - Unclear Risk  No information on how confounding was handled. | - Unclear Risk  Authors did not report any missing data. |
| **Terrell 1993** | - Low Risk  Participants of the pre-post periods belong to the same population. | - Low Risk  “There was a total hospital-wide conversion that did not permit substitution”  “Classes were held every 30 minutes, 16 hours a day for a week to enable all shifts of all departments to participate”  “Liaison nurses from the IV therapy department worked with the nursing units to effect a smooth transition and to in service any nurses who may have missed the scheduled classes” | - High Risk  “If an employee has a NSI they are instructed to report the incident immediately” but no active surveillance or mandatory forms | - Unclear risk  No information on how confounding was handled. | - Unclear Risk  No missing Data mentioned. |
| **Orenstein 1995** | -Low risk  Participants of the pre-post periods belong to the same population | -Low risk  “No study unit was using protective devices before the  study”  “Traditional devices … were removed from all study units”  An infection control practitioner reviewed weekly the number and type of intravenous lines in use on the study units . | -Low risk  Three mechanism used to collect outcome data  “NSIs were actively captured; this decreased the  likelihood of underreporting” | -Low risk  “Control for patient acuity,  number of HCW days and reporting methods” | - Unclear Risk.  Authors did not report any missing data |
| **Hoffman 2013** | -Low risk  Participants of the pre-post periods belong to the same population although the population size increased slightly with time | - Low risk  “Safety devices were introduced throughout the hospital including all departments and all operating rooms”; “training was performed in all departments and was obligatory for all healthcare personnel when the new device was introduced” | - Low risk  “Data was extracted from mandatory needlestick report”. | -Unclear risk  No information on how confounding was handled.  “The study did not cover degree of extended work shifts, time pressure or under-staffing influencing the number of needlestick infections” | - Unclear risk  Authors did not report any missing data |
| **Valls 2007** | -Low risk  Participants of the Pre-Post periods belong to the same population  “We selected fall and winter months, to assure continuity among the employees involved in the study” | -Low risk  “Insulin injection systems (“insulin pens”) were  the only nonsafety needle devices that HCWs were allowed to use”., | -Low risk  “The nurses in charge of the study carried out active surveillance and reporting of injuries during the intervention period.”  Also, educational activities focused on the importance of reporting injuries. | - Unclear risk  Not clear that all confounding variables were controlled for.  “We tried to maintain control of possible prognostic variables involved in the study by selecting the same period of the year and by conducting the study in hospital areas with homogeneously distributed activities” | -Low risk  Authors did not report any missing data |
| **Whitby 2008** | -Low risk  HCW from the same hospital included in the before and after phases of the study  Participation rate: not mentioned | -Low risk  At the commencement of the intervention in 2005, all conventional syringes and needles that were no longer required were physically removed. | -Low risk  The same system of reporting of NSI to the Infectious Diseases Department, which has been in place since 1996, was used throughout the study.  All data pertaining to this study were collected prospectively | -Unclear risk  No information on how confounding was handled. | - Unclear risk  Authors did not report any missing data |
| **Sohn 2004** | -Low risk  Participants from the same hospital in the pre and post period | - Low risk  Prior to the program’s implementation, less than 2% of devices in use were safety devices | -Low risk  Prospectively collected data on needle stick injuries | -Unclear risk  No information on how confounding was handled. | -Unclear risk  Authors did not report any missing data |
| **Wolfrum 1994**  ***A follow-up evaluation to a needle-free IV system*** | -Low risk  Participants of the pre-post periods belong to the same population.  Participation rate: not mentioned | -High risk  Investigators do not report removal of all conventional devices from the experimental wards. Also, there is a risk of contamination given that not all hospital wards were included in the intervention: “..Some of the hospital areas do not or cannot use the needle free system”  “In 1991 the hospital instituted a needle-free system in addition to employee and product educational programs” | -Unclear risk  No specific data on how NSIs were reported: “Employees within the institution reported exposure.”  “A follow up survey on the needle-free system was conducted in 1993” (after the study period) “and send to all RNs using the system, 38% of RNs respond to the survey.” | - Unclear Risk  No information on how confounding was handled. | - Unclear Risk  Authors did not report any |
| **Gershon 1999** | -Low risk  Participants from the same hospital | -Unclear risk  “HCWs from the same hospital  over the course  of 2 months, all alternative systems were phased out”  “lack of control over secular trends at the hospital” (contamination bias) | -Low risk  “reliance on  self-reported injuries, which raises the issue of underreporting  and selection bias.”  “Recall bias was limited by having the injured workers complete  the questionnaire at the time they reported the injury”  “Reporting was mandatory for hospital employee and incentives were provided to encourage reporting”. | -Unclear risk  “One important limitation of the study was the difficulty  in determining the impact of the individual interventions,  since several interventions were implemented concurrently.”  No controlling for this confounder reported. | -High risk  Pre intervention: 1 missing  Post intervention: 6 missing. |
| **La Montagne 2007** | -High risk  “The comparability  between the 2 surveys could also be an issue,  since participating hospitals and medical units were not strictly identical.” | -High risk  No data reports that conventional devices were removed from services at the beginning of the study.  No training reported.  The only definition  is a functional one (drawn from manufacturers’ safety-engineered  modifications of invasive devices) and is not entirely evidence based.” | -Unclear risk  “Possible underreporting of occupational injuries since they are self reported”  “To avoid this possible  bias, the annual number of procedures performed by participating  nurses was indirectly calculated.”  “the analyses lacked statistical power, given the low number  of NSIs reported. Thus, the SED impact could not be  assessed for every type of device.” | -Unclear risk  “the classification  of devices into 2 categories (safe and nonsafe) is  somewhat arbitrary, since the notion of safety is relative. No  official list of SEDs exists in any country.  “the units purchasing more SEDs might also provide more  prevention training to their staff), which may have biased the  device impact measures.” | -Unclear risk  No loss of data reported. |
| **McCleary 2002** | -Low risk.  Participants selected from the same population in the pre and post period | -Low risk  Criterion used to select study group: use of unguarded AVFN in these clinics prior to the study  “training in the proper use of the guarded AVFN was added to the mandatory training for the prospective period”  “Training was given to HCWs newly hired during the study prior to their use of the guarded AVFN, as well as to anyone requesting additional training” | -Unclear risk  Criterion used to select study group: “preexistence of a system within the clinics for reporting NSIs, availability of historical data from the participating clinics”  However, no information given on how the NSI was reported. | - Unclear Risk  No information on how confounding was handled. | - Unclear Risk  Authors did not report any missing data. |
| **Mendelson 1997** | -Low risk.  Participants selected from the same population in the pre and post period with a marked decrease in their number in the intervention period | -Low risk.  “Investigators replaced conventional phlebotomy devices with safety devices  Hospitalwide, monitored supplies of phlebotomy equipment to attempt to ensure that only safety devices were available… and inventoried the autoclaved contents of a representative sample of disposal  containers for sharp instruments to determine rates of use of safety devices and conventional devices and rates of activation of safety features” | -Low risk.  Surveys were used to estimate under-reporting (by self) | - Unclear Risk  No information on how confounding was handled. | - Unclear Risk  No missing Data mentioned. |
| **Adams 2006** | -Low risk.  Participants selected from the same population in the pre and post period | - Low risk  “Standard needles were removed except for the cardiac arrest trolley, as members of the cardiac arrest team may not all have been familiar with the safety devices”  “ On completion of the training programme, the safety needles were introduced into the clinical areas” | -Low risk  “Prospective NSI data were captured from 2001 onwards from reports to the Trust’s occupational health and safety department and risk management” | -Unclear risk  No information on how confounding was handled.  We (systematic review team) attempted to minimize confounding by focusing on the data from 2001 and 2004 | - Unclear risk  Authors did not report any missing data |
| **Rogues 2004**  ***Impact of safety devices for preventing***  ***percutaneous injuries related to phlebotomy procedures in health-care workers*** | -Low risk  Participants  from same hospital during the pre and post intervention period. | - Low risk  “Conventional phlebotomy nonsafety devices were removed from all departments, and the new products were in place on implementation day.” | -Unclear risk  Data was prospectively reported.  “reported needlestick injuries, which can be biased if HCWs overreport or underreport injuries during the 2  Periods” | -High risk  “the lack of data on  compliance with device activation”  “Evidence  that reductions of needlestick injury rates are directly attributable to a safety device alone is scarce.”  “Injuries that occur despite implementation of a  safety device may be due to mechanical failure of the  safety feature, failure of activation by not attempting or  not completing activation, or an inherent risk in the activation procedures” | -High risk  Missing data during the pre-intervention years 1993-1994 about the estimated number of phlebotomies  Performed. |
| **Billiet 1991** | -Low risk  Participants from the same phlebotomy laboratory | -Unclear risk  No information about timeliness and completeness of removal of standard devices and introduction of new devices | -Unclear risk  No information provided on how NSI events were collected (e.g., use of a mandatory registry) | -Unclear risk  No information on how confounding was handled. | -Unclear risk  No missing data reported |
